# A cadherin-integrin–ECM code for presomitic mesoderm fluidity

**DOI:** 10.1242/dev.204874

**Published:** 2025-11-03

**Authors:** Miriam A. Genuth, Dörthe Jülich, Andrew T. Ton, Sarah J. Smith, Emilie Guillon, Mark D. Shattuck, Corey S. O'Hern, Scott A. Holley

**Affiliations:** ^1^ Department of Molecular, Cellular and Developmental Biology, Yale University, New Haven, CT 06520, USA; ^2^ Department of Physics, Yale University, New Haven, CT 06520, USA; ^3^ Department of Physics and Benjamin Levich Institute, City College of New York, New York, NY 10031, USA; ^4^ Department of Mechanical Engineering, Yale University, New Haven, CT 06520, USA

**Keywords:** Cadherin, Integrin, Fibronectin, Fibrillin, Zebrafish

## Abstract

Animal tissues exist within a continuum of fluid to solid states, and transitions between states are important for embryonic development, wound healing and cancer metastasis. Fluid-to-solid transitions are governed by the ratio of adhesive energy to kinetic energy. Here, we find that presomitic mesoderm solidification is driven by an intrinsic decline in cell speed along with an increase in adhesion mediated by Cadherin 2 in parallel with fibronectin and its receptor Integrin α5. A computational model of cell–cell adhesion in the central tissue mesenchyme and cell–ECM adhesion on the tissue surface explains the observed phenotypes. Further, we identify negative feedback within the ECM as fibronectin supports the formation of a separate layer of Fibrillin 2b matrix that inhibits solidification. These data reveal a tissue fluidity code in which solidification is promoted by cadherins in parallel with Integrin α5 and fibronectin, whereas negative feedback through Fibrillin 2b promotes fluidization.

## Teaser

**Summary:** Analysis of compound mutants and 2D computational modeling reveals a tissue fluidity code whereby solidification is promoted by Cadherin 2 in parallel with Integrin α5 and fibronectin, whereas negative feedback through Fibrillin 2b promotes tissue fluidization.

## INTRODUCTION

**Fig. 1.**
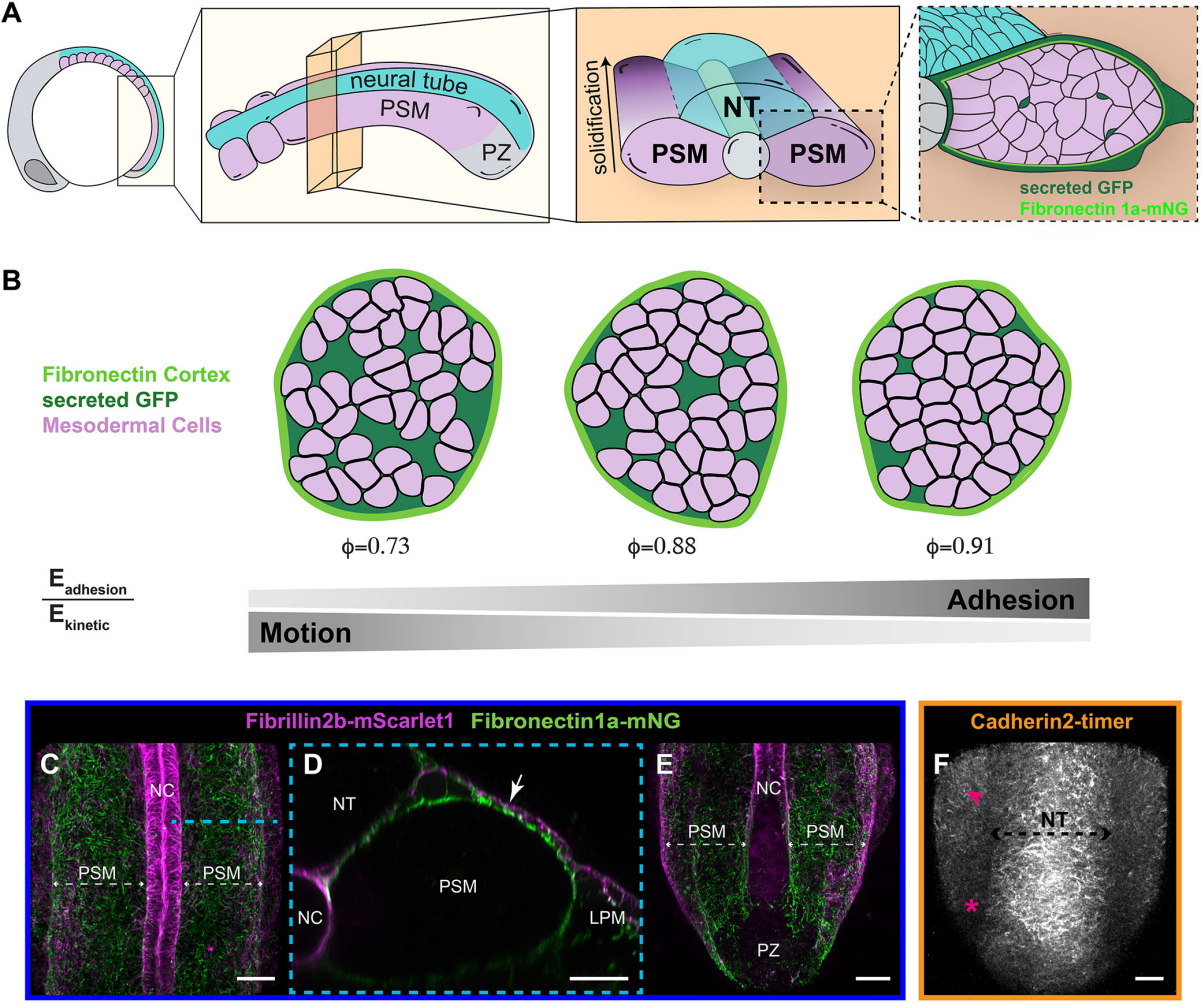
**Solidification of the presomitic mesoderm.** (A) Illustration of the solidifying presomitic mesoderm (PSM) in a 10-somite-stage zebrafish embryo. From left to right is the sequential magnification of the posterior body. The two boxes on the right show transverse sections. PSM cells are pink, neural tube (NT) cells are cyan. The PSM is coated by fibronectin (light green) and the extracellular space is highlighted by secreted GFP (dark green). (B) 2D Simulations of PSM solidification show the fibronectin tissue cortex in light green, secreted GFP in dark green, and PSM cells in pink. As adhesion energy increases relative to kinetic energy, the packing fraction φ (i.e. the ratio of the cellular area to the tissue area) increases. (C-E) Confocal images showing localization of endogenously tagged Fibrillin 2b (in magenta) and Fibronectin1a (in green) in the tailbud. (C) The PSM is coated in an ECM of fibronectin and Fibrillin 2b. Fibrillin 2b is prominent in the notochord (NC) ECM (*n*=12). (D) Transverse section of the area highlighted by the cyan dashed line in C. Fibrillin 2b localizes dorsally atop fibronectin on the PSM (white arrow). (E) Neither Fibrillin 2b nor fibronectin is assembled into a matrix within the progenitor zone (PZ). There is a fourfold increase in ECM intensity comparing 50 µm^2^ regions in the mid-PSM and PZ (*n*=3). (F) Cadherin 2 adhesions stabilize gradually from posterior (pink asterisk) to anterior (pink arrowhead) PSM. Cadherin 2 is tagged in tandem with the slower maturing TagRFP (maturation time 100 min) and sfGFP (maturation time 13.6 min) [TgBAC(*cdh2:cdh2-tFP*)]. Only TagRFP (in grayscale) is shown [*n*=18; mean intensity value for TagRFP in a 30 µm^2^ centered on the arrowhead area is 1.94-fold greater than the area around the asterisk, compared to sfGFP with a fold change of 1.66 (paired *t*-test, *P*=0.0063)]. Images in C,E,F are 3D projections of confocal *z*-stacks with anterior to the top. Scale bars: 30 µm (C,E,F); 15 µm (D). LPM, lateral plate mesoderm. See also Fig. S1.

The tailbud is the posterior end of the elongating vertebrate embryo and is largely composed of the posterior neural tube, notochord and paraxial mesoderm (Fig. 1A). These tissues give rise to the spinal column, dermis and musculature of the trunk and tail. Just beneath the posterior-most epidermis, neuromesodermal progenitors expressing *brachyury* (*tbxta*) and *sox2* will differentiate as either neural tube or paraxial mesoderm progenitors (Martin and Steventon, 2022). The mesodermal progenitors express *brachyury* and *tbx16*, and their progenitor zone (PZ) behaves as a fluid with rapid, disorderly cell motion and frequent cell neighbor exchanges (Das et al., 2017; Lawton et al., 2013). Cells leave the PZ and join the posterior end of the left and right presomitic mesoderm (PSM) where the cell velocity drops precipitously (Benazeraf et al., 2010; Lawton et al., 2013). Cell velocity continues to slow as the PSM matures and solidifies. In parallel with cell deceleration, the paraxial mesoderm exhibits a rigidity gradient in which the PZ is softer than the posterior PSM, which is in turn less rigid than the anterior PSM (Mongera et al., 2018). These mechanical changes have been ascribed to a fluid-to-solid jamming transition (Mongera et al., 2018).

A jamming transition occurs when a disordered particulate material changes from behaving like a fluid to behaving like a solid when the density of the material exceeds a critical packing fraction. The packing fraction is the ratio of the volume of the particles to the total volume of the system, or, in the case of the PSM, the ratio of the cellular volume to the tissue volume. In the PSM, the packing fraction increases as the tissue matures and there is less extracellular space between cells (Fig. 1A) (Mongera et al., 2018). A familiar example of a jamming transition is the cessation of the flow of salt from a shaker. In the fluid state, the salt particles flow freely and exchange neighbors. When jammed, the salt particles are locked in place. During the jamming transition the system gains non-zero shear modulus, enabling it to withstand shear deformation (O'Hern et al., 2003). The critical packing fraction depends on the details of the system and has been calculated for various combinations of temperature, shear, particle shape, deformability, and adhesion/repulsive strength (Bi et al., 2015; Boromand et al., 2018; Koeze and Tighe, 2018; Liu and Nagel, 2010; O'Hern et al., 2003). Two particularly salient parameters for biological systems are the kinetic and adhesive energies of the cells. Kinetic energy is an analog for temperature and represents the intrinsic velocity fluctuations of the cell. The adhesive energy quantifies the strength of cell–cell and cell–extracellular matrix (ECM) attachments. The ratio of kinetic and adhesive energies influences the packing of the tissue with high kinetic energy promoting fluidization and high adhesive energy promoting jamming behavior (Fig. 1B).

A jamming transition requires physical constraints on the system (Araújo et al., 2023). The PSM has two notable constraints: cell–cell adhesion and cell–ECM attachments along the tissue boundary. Prior studies found that cell motion is more disordered in the PSM of mutants of the cell adhesion protein Cadherin 2 (*cdh2^−/−^*), and tissue stiffness is reduced indicating a role for *cdh2* in solidification (Lawton et al., 2013; Mongera et al., 2018). The PSM also requires Integrin α5β1 to assemble a fibronectin-ECM sheath that surrounds the tissue and mediates adhesion with adjacent tissues (Fig. 1C-E) (Dray et al., 2013; Guillon et al., 2020; Jülich et al., 2015). Thus, the PSM creates its own exterior boundary. However, concomitant knockdown of the two main fibronectin receptors did not alter cell motion in the zebrafish PSM and inhibiting fibronectin fibrillogenesis in the *Xenopus* PSM did not prevent tissue stiffening, suggesting no requirement for cell–fibronectin adhesion in PSM solidification (Dray et al., 2013; Zhou et al., 2009).

The ECM is a composite of proteoglycans, and we sought other ECM proteins that may regulate PSM fluidity. Fibrillin 2 is a strong candidate as it is an ECM protein that localizes to the surface of the paraxial mesoderm of quail embryos (Czirok et al., 2004). Fibrillin 2 is a major component of elastic microfibrils, and phylogenetically predates fibronectin (Halper and Kjaer, 2014). Like fibronectin, Fibrillin 2 is an Integrin α5β1 ligand, and Fibrillin 2b was isolated via co-immunoprecipitation mass spectrometry of Integrin α5β1 in zebrafish embryos (Sun et al., 2021). Zebrafish *fibrillin 2b* mutants exhibit notochord, vascular and fin fold defects (Gansner et al., 2008). In humans, Fibrillin 2 variants are associated with severe kyphoscoliosis among other features of a congenital connective tissue disorder (Robinson et al., 2006).

Here, we take a systems approach to re-examine the role of cell–cell and cell–ECM adhesion in zebrafish PSM solidification. We generated new knockout alleles of *fibrillin 2b* as well as a fully functional fluorescent protein knock-in allele to visualize Fibrillin 2b ECM in live embryos. We examined single, double, triple and quadruple loss-of-function phenotypes to reveal genetic redundancy and negative feedback that governs PSM solidification. Using live imaging and machine learning-aided data analysis, we systematically quantified the tissue packing fraction, cell shape, cell speed and cell neighbor exchanges. We compare these results to those from a 2D computational model of tissue solidification. Lastly, we examined regulation within the ECM by quantifying ECM morphology via live imaging of wild-type and mutant embryos and by measuring Integrin α5β1 activation using an *in vivo* FRET-FLIM (fluorescence resonance energy transfer by fluorescence lifetime imaging) assay. These experiments identify a tissue fluidity code in which solidification is promoted by Cadherin 2 in parallel with Integrin α5 and fibronectin, whereas negative feedback through Fibrillin 2b promotes fluidization.

## RESULTS

### ECM assembly and Cadherin 2 stabilization during PSM maturation

We previously generated functional fluorescent protein knock-in alleles of *fibronectin 1a* and *fibronectin 1b* to enable live imaging of the ECM (Jülich and Holley, 2024). Here, we tagged endogenous Fibrillin 2b by inserting mScarlet1 within a flexible region just after a Furin cleavage site near the N terminus of the protein (Fig. S1A) (Jensen et al., 2014). This knock-in allele complements the *fbn2b* (also known as *fbn3*) mutant allele indicating that the knock-in retains function (Fig. S1). Combining the Fibrillin-2b-mScarlet1 transgene with a Fibronectin-1a-mNG knock-in allele, we observed an ECM consisting of fibronectin and Fibrillin 2b that coats the surface of the paraxial mesoderm (Fig. 1C,D), but the ECM was missing in the fluid mesodermal PZ in the posterior tailbud (Fig. 1E). Thus, there is a positive correlation between the presence of ECM and tissue solidification.

To examine the distribution of cell–cell adhesions, we used a Cadherin 2 tandem fluorescent timer transgenic zebrafish expressing Cadherin 2 tagged with both a fast-maturing superfolder (sf)GFP and a slow-maturing TagRFP (Revenu et al., 2014). Only stable Cadherin 2 proteins exhibit RFP fluorescence, and they are enriched in cell–cell adhesions (Cavey et al., 2008; Fagotto et al., 2013; Revenu et al., 2014). We previously found that the Cadherin 2 timer transgene reports a decline in cell adhesions as neuromesodermal progenitors enter the fluid PZ domain (Das et al., 2017). Here, we observed that the Cadherin 2 timer transgenic exhibits increased RFP fluorescence as PZ cells assimilate into the posterior PSM (Fig. 1F). Therefore, cell–cell adhesions with stable Cadherin 2 accumulate as the PSM solidifies.

### Tissue packing fraction and cell shape

**Fig. 2.**
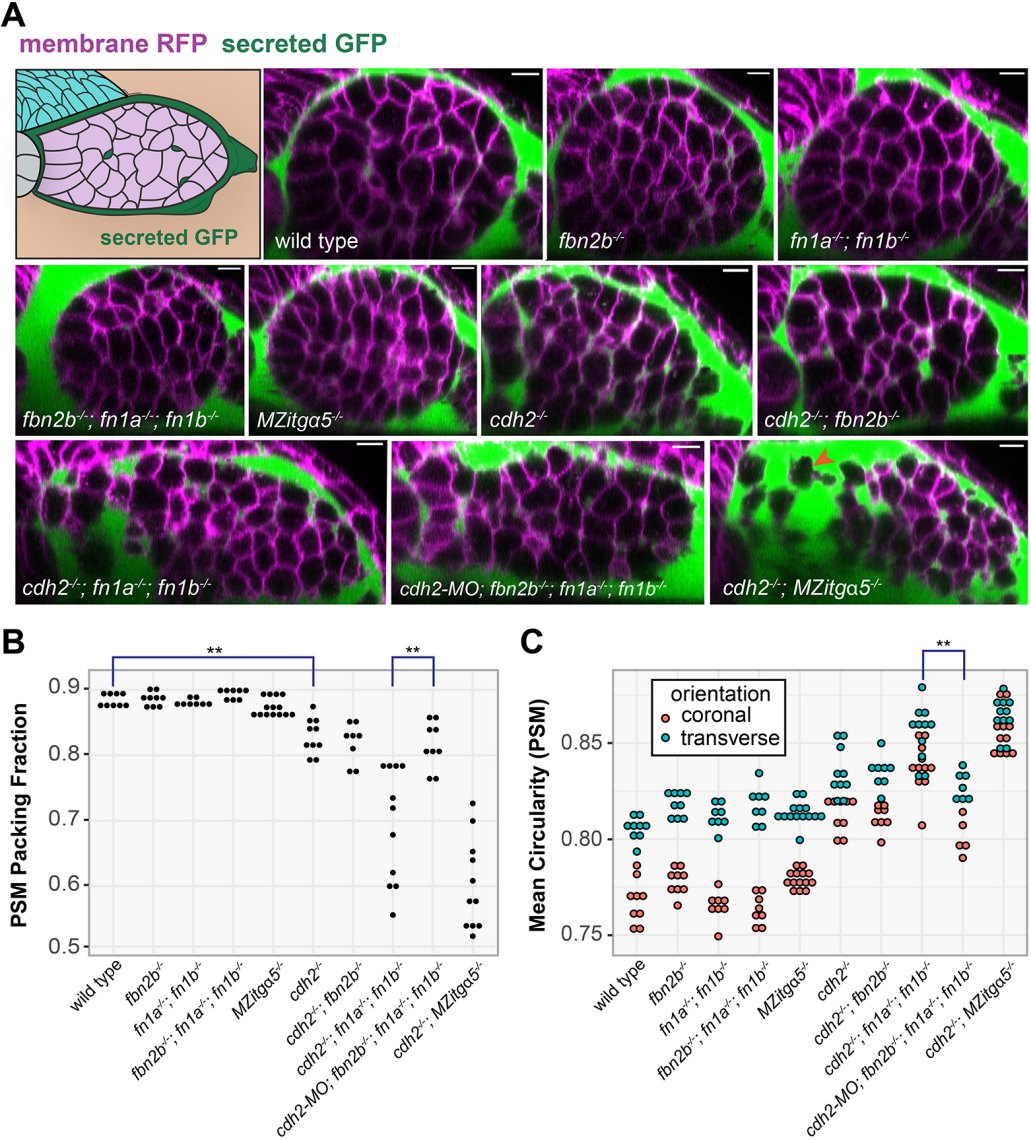
**PSM tissue packing and cell shapes.** (A) Graphical illustration (top left) and transverse slices within the mid-PSM of confocal images of embryos expressing membrane RFP (magenta) and secreted GFP (green). The orange arrowhead in the lower right panel indicates a cell with only one neighbor. Scale bars: 10 µm. (B) PSM packing fraction of individual PSMs. The packing fraction in *cdh2^−/−^* is reduced compared to wild type (unpaired, two-way, two-tailed Student's *t*-test, **P<0.01), and the packing fraction in *cdh2-*MO*; fbn2b^−/−^; fn1a^−/−^; fn1b^−/−^* is higher than in *cdh2^−/−^; fn1a^−/−^; fn1b^−/−^* (unpaired, two-way, two-tailed Student's *t*-test, **P<0.01). Each black dot represents one PSM analyzed. Statistics calculated with multiple testing corrections. See also Table S2A. (C) Mean circularity of cells in individual PSMs measured on a scale from 0 to 1 with 1 being a perfect circle. Circularity is measured in both coronal (orange) and transverse (cyan) orientations. Genotypes with high packing fraction (wild type, *fbn2b^−/−^*, *fn1a^−/−^; fn1b^−/−^*, *fbn2b^−/−^; fn1a^−/−^; fn1b^−/−^* and *MZitgα5^−/−^*) exhibit different cells shapes in transverse and coronal planes. Loss of *fibrillin2b* in *cdh2-*MO*; fn1a^−/−^; fn1b^−/−^* rescues cell morphology compared to *cdh2^−/−^; fn1a^−/−^; fn1b^−/−^* (unpaired, two-way, two-tailed Student's test, **P<0.01). Each orange and cyan dot represents one PSM analyzed. See also Figs S2-S5 and Table S2B for detailed statistical analysis.

To study the role of cell-cell and cell-ECM adhesion in tissue packing, we used secreted GFP to label the extracellular space and a membrane RFP to label the cell cortex (Fig. 2A). We imaged the tissue in 3D in live embryos and calculated the packing fraction within the PSM, i.e. the ratio of cellular volume to total tissue volume. We quantified packing fraction in every 2D transverse slice along the anterior-posterior axis of the PSM. The PSM area was defined for each transverse slice as the convex hull around the GFP-negative region, excluding any intrusions of other tissues. We measured cell circularity in both transverse and coronal 2D slices spaced every 5 µm along the anterior-posterior or dorsal-ventral axes, respectively. Both the packing fraction and cell shape measurements produced very consistent distributions for PSMs within genotypes (Figs S2, S5).

Confirming the role of Cadherin 2 in tissue solidification, we found that the packing fraction of the PSM is reduced from 0.89 in wild type to 0.83 in *cdh2^−/−^* (Fig. 2A,B, Fig. S2). We reassessed the role of cell–ECM adhesion in PSM solidification by generating double and triple mutants with *cdh2^−/−^*. Consistent with prior results, we observed solid wild type-like packing fractions in maternal-zygotic *itga5* mutants (*MZitgα5^−/−^*) and embryos double mutant for the two zebrafish fibronectin genes (*fn1a^−/−^; fn1b^−/−^*). However, *cdh2^−/−^; MZitgα5^−/−^* double mutants and *cdh2^−/−^; fn1a^−/−^; fn1b^−/−^* triple mutants have a synergistic decrease in packing fraction to 0.60 and 0.70, respectively (Fig. 2B). The posterior PSM is wide in the medial-lateral axis, and it adopts a circular shape in transverse section as it matures. The anterior PSM remains wide medial-laterally in *cdh2^−/−^; MZitgα5^−/−^* double mutants and *cdh2^−/−^; fn1a^−/−^; fn1b^−/−^* triple mutants, suggesting that increased adhesion drives this change in shape by minimizing the surface to volume ratio. Overall, these results indicate that prior experiments assessing the role of Integrin α5-fibronectin adhesion in PSM solidification were confounded by genetic redundancy. Our results indicate that Cadherin 2 and Integrin α5-fibronectin function in parallel to promote PSM solidification.

The stronger phenotype of the *cdh2^−/−^; MZitgα5^−/−^* double mutants relative to the *cdh2^−/−^; fn1a^−/−^; fn1b^−/−^* triple mutants suggests a role for another Integrin α5 ligand. We examined the function of the ECM protein and Integrin α5 ligand Fibrillin 2 by generating new knock-out alleles (Fig. S3). Surprisingly, *fibrillin 2b* opposes tissue solidification. The PSM packing fraction of *fbn2b^−/−^* mutants was similar to that of wild type, and loss of *fibrillin 2b* did not enhance the *cdh2^−/−^* decrease in packing fraction (Fig. 2A,B, Fig. S2). In fact, in the absence of the fibronectin genes, loss of *fibrillin 2b* increased the packing fraction: *fbn2b^−/−^; fn1a^−/−^; fn1b^−/−^* had a higher packing fraction than wild type. Strikingly, the packing fraction of *cdh2^MO^; fbn2b^−/−^; fn1a^−/−^; fn1b^−/−^* increased to 0.82 compared to 0.70 in *cdh2^−/−^; fn1a^−/−^; fn1b^−/−^* and resembled that of *cdh2^−/−^* single mutants. Therefore, Integrin α5 has three ECM ligands that regulate the PSM packing fraction. Fibronectin 1a and Fibronectin 1b promote PSM solidification whereas Fibrillin 2b promotes fluidization.

Cells in densely packed solid tissues adhere to each other and adopt polyhedral shapes whereas cells in more disperse fluid tissues exhibit more spherical shapes. We systematically quantified cell shape in 2D in both coronal and transverse slices of the PSM (Fig. 2C, Figs S4 and S5). In wild-type and genotypes with high packing fractions, such as *fbn2b^−/−^*, *fn1a^−/−^; fn1b^−/−^*, *fbn2b^−/−^; fn1a^−/−^; fn1b^−/−^* and *MZitgα5^−/−^*, cells were less circular and exhibited different cell shapes in transverse and coronal planes. This latter result reveals differences in cell polarity along the transverse and coronal planes. In genotypes with lower packing fractions of 0.83-0.82, such as *cdh2^−/−^*, *cdh2^−/−^; fbn2b^−/−^* and *cdh2^MO^; fbn2b^−/−^; fn1a^−/−^; fn1b^−/−^*, cell circularity increased, and cell polarity therefore diminished. In the two genotypes with the lowest packing fractions of 0.70 and 0.60, *cdh2^−/−^; fn1a^−/−^; fn1b^−/−^* and *cdh2^−/−^; MZitgα5^−/−^*, respectively, circularity increased further, and there were no differences in circularity between coronal and transverse slices, indicating that the cells are more spherical in shape. Again, loss of *fibrillin 2b* had the opposite effect as loss of fibronectin genes. Comparing *cdh2^MO^; fbn2b^−/−^; fn1a^−/−^; fn1b^−/−^* to *cdh2^−/−^; fn1a^−/−^; fn1b^−/−^*, loss of *fibrillin 2b* rescued cell morphology to less circular shapes, indicative of improved solidification. Overall, tissue packing fraction decreased as cell circularity increased across genotypes. These morphometric data suggest that Cadherin 2 promotes PSM solidification in parallel with Integrin α5 and fibronectin, whereas Fibrillin 2b promotes fluidization.

Note that we used a *cdh2* morpholino (MO) for the quadruple loss-of-function experiments. We compare the *cdh2^−/−^* mutant and the *cdh2^MO^* phenotypes in Figs S2 and S5 and Table S2. The *cdh2* morpholino quantitatively phenocopied the changes in PSM packing fraction, cell shape in transverse sections and cell motion observed in the *cdh2* mutant. Only cell shape in coronal sections showed a difference in circularity of 0.81 in *cdh2^−/−^* compared to 0.80 in *cdh2^MO^* (*P*=0.01347).

### Cell speed and cell mixing

**Fig. 3.**
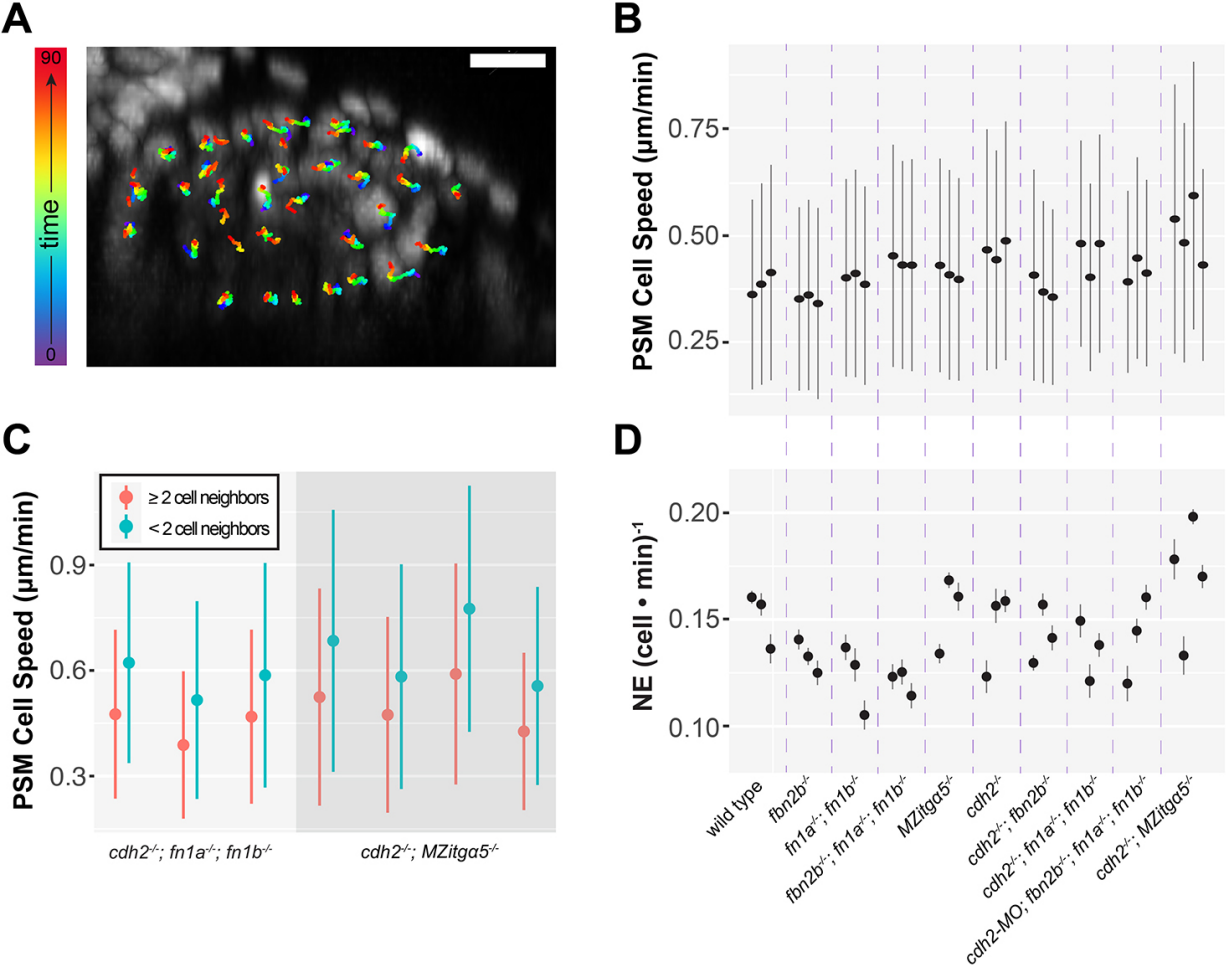
**Cell motion in the PSM.** (A) Representative transverse image of cell tracks in a 90 min timelapse in the PSM of a wild-type embryo from time t=0 (purple) to 90 min (red). Scale bar: 10 µm. (B) Mean and s.d. of cell speed in individual embryos (*n*=3 for each genetic background except *cdh2^−/−^; MZitgα5^−/−^* with *n*=4). (C) Speed of isolated cells (zero or only one neighbor) and those with two or more neighbors in individual *cdh2^−/−^; fn1a^−/−^; fn1b^−/−^* (*n*=3) and *cdh2^−/−^; MZitgα5^−/−^* (*n*=4) embryos. Cell speeds are higher in cells with fewer than two neighbors in both intra-embryo comparisons (unpaired, two-way, two-tailed Student's *t*-test) and averages (paired *t*-test, *P*<0.001). (D) Cell neighbor exchange rates in the same individual embryos as in B. The consistent trend of slightly increased speeds and cell mixing in the bulk cell movement data in *cdh2^−/−^*, *cdh2^−/−^; fn1a^−/−^; fn1b^−/−^* and *cdh2^−/−^; MZitgα5^−/−^* embryos are not statistically significant. For detailed statistical analysis, see Table S2C.

Having identified morphological changes consistent with alteration of tissue fluidity, we next examined tissue dynamics by performing 3D time-lapse imaging and tracking cell motion in *x*, *y* and *z* planes in the PSM (Fig. 3A). Cells should increase both their speed and rate of cell neighbor exchange in more fluid tissues. Indeed, we observed slightly increased cell speed in *cdh2^−/−^; MZitgα5^−/−^* embryos (Fig. 3B, Table S2C).

To determine whether crowding affects cell speed, we identified isolated PSM cells with fewer than two neighbors from *cdh2^−/−^; fn1a^−/−^; fn1b^−/−^* and *cdh2^−/−^; MZitgα5^−/−^* embryos (Fig. 2A, lower right panel, orange arrowhead). We compared the speeds of these isolated cells to those of the bulk population of the PSM. These cells made up 3.8-5.7% of the PSM in these genotypes, and they moved significantly faster than the rest of the population, indicating that a high packing fraction does restrict cell motion in the PSM (Fig. 3C). Note that the absolute speeds varied from embryo to embryo, but the differences in speed between the isolated cells and the bulk cells was relatively consistent. However, these isolated PSM cells were no faster than PZ cells even though they were present at a much lower packing fraction than in the PZ (Lawton et al., 2013; Mongera et al., 2018). Wild-type cells reduced their speeds by 0.25 µm/min at the PZ-to-PSM transition, but isolated cells in the *cdh2^−/−^; fn1a^−/−^; fn1b^−/−^* and *cdh2^−/−^; MZitgα5^−/−^* embryos increased their speeds by 0.14 µm/min relative to the bulk population (*P*<0.0001). Thus, jamming does not fully account for the decline in cell speed during PSM solidification. These data suggest that there is also a decline in speed during PSM differentiation that is independent of jamming.

We measured cell neighbor exchanges using a Delaunay triangulation of successive time points within the 4D image datasets of the tissues. However, we did not observe increased cell neighbor exchange. Although the most severe phenotype in our other assays, *cdh2^−/−^; MZitgα5^−/−^* double mutants, exhibited elevated cell neighbor exchange consistent with its fluid morphology (Fig. 3D), this difference was not statistically significant.

To summarize, we observe increased cell speed in *cdh2^−/−^; MZitgα5^−/−^* double mutants in the bulk cell movement data. The intra-embryo comparison between isolated PSM cells and cells with two or more neighbors indicates a density-dependent suppression of cell motion during PSM solidification. Overall, the changes in cell dynamics are smaller than the alterations of PSM packing fraction and cell shape after reduction in cell–cell and cell–ECM adhesion. We conclude that cell–cell adhesion and cell–ECM adhesion function in parallel to promote tissue solidification in conjunction with a decline in speed during PSM differentiation that is independent of jamming.

### A 2D computational model of PSM solidification

Our *in vivo* datasets are 3D for cell packing and cell shape and 3D plus time for cell movement. We also analyzed cell speed and cell neighbor exchange data in 3D. However, it is computationally more straightforward to measure packing fraction and cell shape in 2D transverse sections. Similarly, it is more computationally efficient to model tissue solidification in 2D, and we also wanted to use the same methods for measuring packing fraction and cell shape for both the *in vivo* data and simulations.

**Fig. 4.**
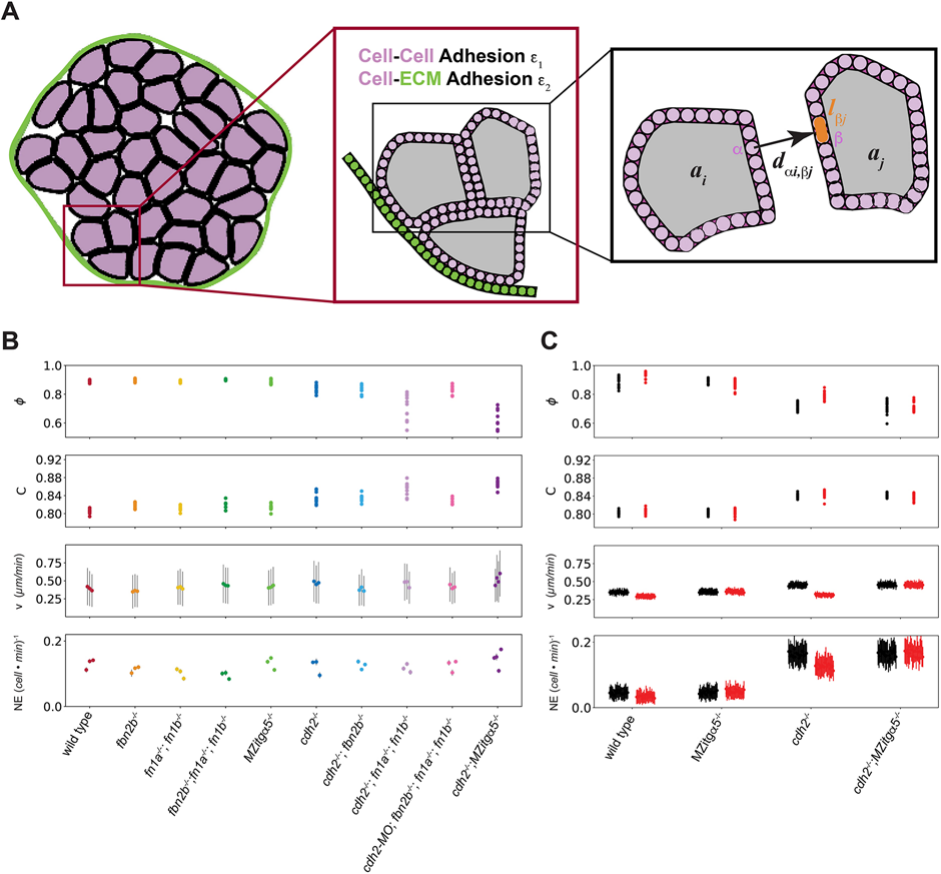
**Discrete element method (DEM) simulations of PSM solidification.** (A) DEM simulations of the deformable particle model (DPM) in 2D for a transverse section of the PSM. In the DPM, cells (pink) are surrounded by an ECM layer (green). Insets illustrate that cell–cell and cell–ECM adhesion strengths are specified by ε_1_ and ε_2_, respectively. Each cell is represented by a deformable particle, consisting of a polygon with 30 vertices (labeled by α and β). Intercellular forces are a function of *d*_αi,βj_, which is the shortest distance between vertex α and the line segment *l*_βj_ between vertices β and β+1 on cell j (Fig. S6). (B,C) Mean packing fraction φ, mean cell circularity *C*, mean cell speed *v*, and mean neighbor exchange *NE* for each of the experimental genotypes (B) and several random initial conditions in the simulations (C). Each point represents the average over an individual embryo or random initial condition, and the standard deviation is indicated for the mean cell speeds and mean neighbor exchanges. Results from simulations with and without Cadherin 2 inhibition of Integrin α5 are shown in red and black, respectively. See also Fig. S6, Movies 1 and 2 and Table S1.

To test our understanding of the mechanism of solidification of the PSM, we developed and carried out discrete element method simulations of the deformable particle model (DPM) in 2D. The model describes a transverse section of PSM and includes cell–cell adhesion and cell–ECM adhesion on the tissue surface (Fig. 4A, Fig. S6, Table S1). By varying the relative strength of the adhesion parameters, the DPM recapitulated the phenotypes as three broad classes that parallel the experimental results: solid (wild type, *fbn2b^−/−^*, *fn1a^−/−^; fn1b^−/−^*, *fn1a^−/−^; fn1b^−/−^; fbn2b^−/−^* and *MZitgα5^−/−^*), less solid (*cdh2^−/−^*, *cdh2^−/−^; fbn2b^−/−^* and *cdh2^MO^; fn1a^−/−^; fn1b^−/−^; fbn2b^−/−^*) and fluid (*cdh2^−/−^; fn1a^−/−^; fn1b^−/−^* and *cdh2^−/−^; MZitgα5^−/−^*) (Fig. 4B,C).

One discrepancy between the results from the DPM simulations and experiments is the elevated cell neighbor exchanges in the less-solid phenotypes in the simulations (Fig. 4B,C, Movie 1). One explanation for this difference is that we do not specify an intrinsic decline in cell speed in the simulations. A second reason for the difference is likely a previously observed repression of Integrin α5 activation in the tissue mesenchyme by Cadherin 2. Integrin α5 and Cadherin 2 on adjacent cells form a physical complex that maintains Integrin α5 in the inactive conformation. Loss of Cadherin 2 de-represses the integrin and leads to fibronectin matrix formation in the mesenchyme (Jülich et al., 2015). This ECM could constrain cell motion in the absence of Cadherin 2. Indeed, when we incorporated a cadherin-dependent repression of Integrin α5 in the DPM, the simulation results better matched the experimental data (Fig. 4C, Movie 2). Additional complexity could be added to the simulations but with the risk of over-determining the model. The general concordance between the results from the simple 2D computational model and experiments supports the idea that PSM solidification is driven by Cadherin 2-mediated cell–cell adhesion within the tissue mesenchyme and integrin-fibronectin adhesion on the tissue surface.

### A bilayered ECM regulates PSM fluidity

**Fig. 5.**
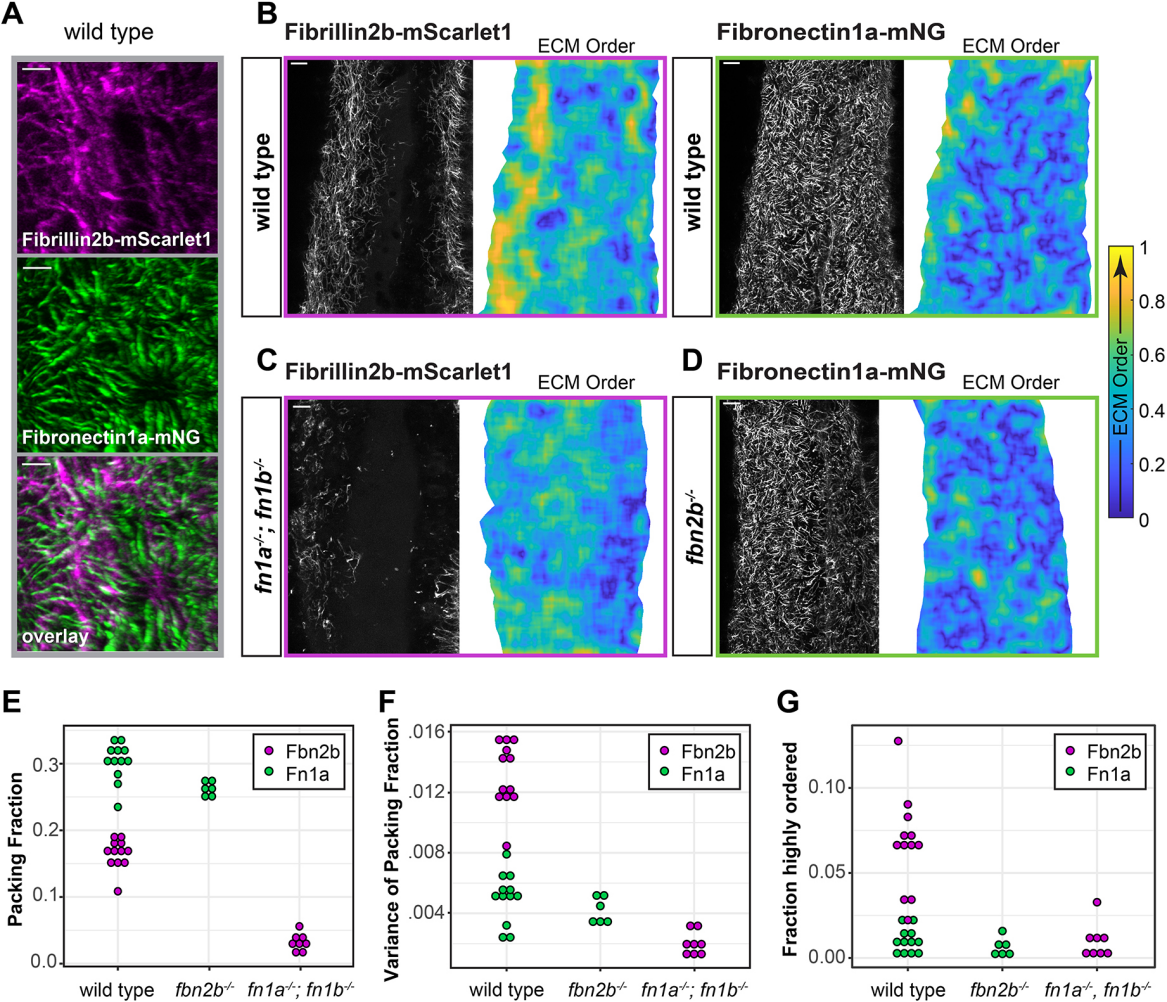
**Organization of the fibronectin and Fibrillin 2b matrix on the PSM.** (A) Super-resolution images of endogenously tagged *fn1a* (green) and *fbn2b* (magenta) on the dorsal PSM surface in wild-type embryos at the 12-somite stage. Scale bars: 10 µm. (B) Super-resolution images and corresponding ECM nematic order showing the alignment of ECM fibers within a 10 µm window in an individual PSM of a wild-type embryo (*n*=12). Warmer colors indicate greater alignment. (C,D) Super-resolution images and ECM nematic order of Fbn2b-mScarlet1 in *fn1a^−/−^; fn1b^−/−^* mutants (*n*=8) (C) and Fn1a-mNG in *fbn2b^−/−^* mutants (*n*=6) (D). (E) Measurements of packing fraction in respective PSMs of the embryos in B-D. Fn1a-mNG is 15% denser in wild type than in *fbn2b^−/−^* mutants (unpaired, two-way, two-tailed Student's *t*-test, *P*<0.01). (F) Variance in packing fraction measured within a 10 µm×10 µm sliding window. (G) Fraction of PSM dorsal surface with highly ordered fibers (nematic parameter≥0.75). Each circle represents one PSM.

We next examined how ECM form and function explain both the role of fibronectin in promoting solidification and the opposing role of Fibrillin 2b in promoting fluidization. Imaging Fibronectin 1a-mNeon Green; Fibrillin 2b-mScarlet1 embryos and larvae revealed distinct and tissue-specific localization patterns of the two ECMs (Fig. S1B-H). Fibronectin 1a and Fibrillin 2b formed distinct fibers in a bilayered ECM ensheathing the PSM with fibronectin closest to the PSM and Fibrillin 2b dorsally atop (Fig. 1C,D, Fig. S1). Fibronectin was often assembled in radial patterns whereas Fibrillin 2b formed parallel fibers (Fig. 5A,B). We characterized the fibronectin and Fibrillin 2b ECMs on the dorsal surface of the PSM by quantifying fiber alignment with a nematic order parameter, packing fraction and variation in packing fraction. Fibrillin 2b was more ordered, meaning that neighboring fibers were more likely to be aligned (Fig. 5B,E). Fibronectin had a higher packing fraction and lower variation in packing fraction, meaning that it was denser and more evenly distributed on the surface of the tissue (Fig. 5F,G).

**Fig. 6.**
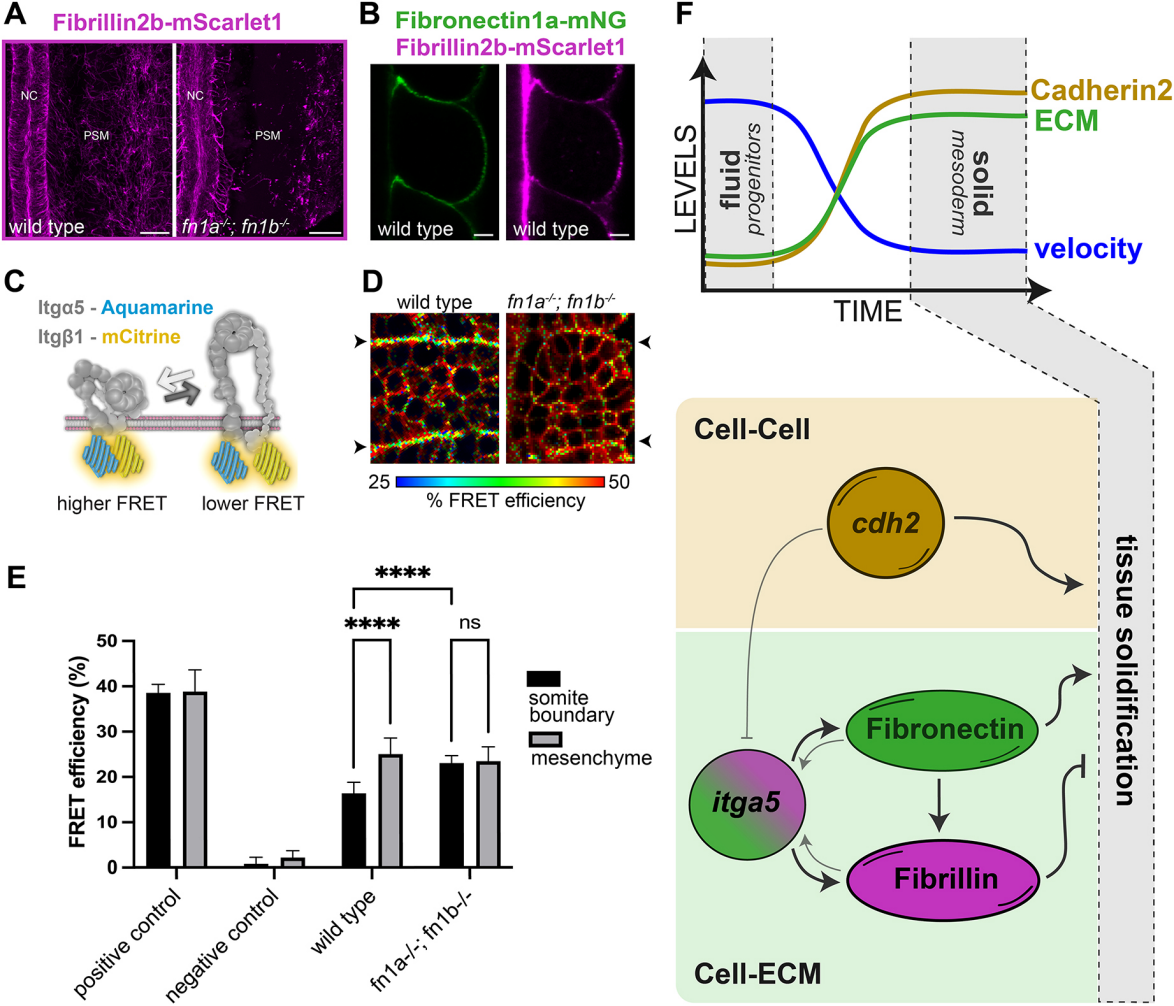
**The ECM and Integrin α5β1 signaling in the paraxial mesoderm.** (A) *fbn2b* on the PSM surface of wild-type (left) and *fn1a^−/−^; fn1b^−/−^* (right) embryos. Scale bars: 20 µm. (B) *fn1a* (left) and *fbn2b* (right) in wild-type embryos localized along somite boundaries. Scale bars: 10 µm. (C) Model illustration of the FLIM-FRET assay for integrin activation. Integrin α5 is tagged with Aquamarine (Aqm) (FRET donor) and Integrin β1a is tagged with mCitrine (mCit) (FRET acceptor). In the inactive conformation, the cytoplasmic tails of Integrin α5 and Integrin β1a are close together leading to high FRET. However, in the active conformation the cytoplasmic tails separate leading to lower FRET. (D) Heatmap for FRET efficiency for Integrin α5-Aqm and Integrin β1a-mCit in wild-type (left) and *fn1a^−/−^; fn1b^−/−^* (right) embryos. Arrowheads indicate somite boundaries. Pixels were binned 5×5. Cooler colors denote lower FRET efficiency, suggesting high levels of integrin activation. (E) FRET efficiency of different integrin pairs: positive control (Integrin α5-Aqm-mCit fusion and Integrin β1a; *n*=7), negative control (Integrin α5-Aqm and Integrin α5-mCit; *n*=8), wild type (Integrin α5-Aqm and Integrin β1a-mCit, *n*=11), *fn1a^−/−^; fn1b^−/−^* (Integrin α5-Aqm and Integrin β1a-mCit, *n*=7). Error bars represent s.d. ****P<0.0001 (one-way ANOVA with Bonferroni correction for multiple comparisons). ns, not significant. (F) Schematic summary of the roles of Cadherin 2, Integrin alpha 5, Fibronectin and Fibrillin 2b in PSM solidification. Top: Solidification of the PSM is preceded by a decline in cell velocity, an increase in stable Cadherin 2 in cell adhesions, and an increase in ECM assembly on the surface of the tissue. Bottom: Cdh2 promotes solidification in parallel with Itgα5 and fibronectin. Cdh2 inhibits Itgα5 to prevent ECM assembly in the PSM mesenchyme. Fibronectin promotes the formation of a Fibrillin 2b ECM, which inhibits tissue solidification. Differential bidirectional signaling between Itgα5 and its two ligands may underlie the different cellular responses to fibronectin and Fibrillin 2b. Paler gray arrows indicate ligand activation of Integrin alpha 5.

Next, we examined cross-regulation between fibronectin and Fibrillin 2b. The Fibrillin 2b matrix was highly dependent on fibronectin genes, but the fibronectin ECM was only slightly less dense in *fbn2b^−/−^* (Fig. 5C-G). This loss of Fibrillin 2b was specific to the PSM as the notochord and lateral plate mesoderm retained their Fibrillin ECM in *fn1a^−/−^; fn1b^−/−^* embryos (Fig. 6A). Thus, in embryos lacking fibronectin genes, the Fibrillin 2b ECM that remained on the notochord and lateral plate likely mediates the fluidizing effect on the PSM. Overall, these data indicate that the Fibrillin 2b matrix functions downstream of the fibronectin matrix on the surface of the PSM to create negative feedback within the ECM that limits tissue solidification.

To better understand the factors that activate Integrin α5β1 in the paraxial mesoderm, we used a FRET-FLIM assay to measure integrin activation (Fig. 6C) (Kim et al., 2003; Sun et al., 2021). In a prior study, comparison of wild-type Integrin α5β1 with a mutant that cannot bind ligand found that Integrin α5β1 is predominantly activated on the tissue boundaries (Sun et al., 2021). Here, we sought to determine whether there are other ECM ligands that activate Integrin α5β1 in the paraxial mesoderm. Specifically, since Fibrillin 2b fiber formation is dependent on fibronectin on the surface of the paraxial mesoderm, then *fn1a^−/−^; fn1b^−/−^* double mutants should eliminate integrin activation on paraxial mesoderm boundaries if there are no fibronectin-independent ligands in the tissue.

We tagged the cytoplasmic tail of Integrin α5 with a FRET donor fluorophore and tagged the cytoplasmic tail of Integrin β1a with a FRET acceptor (Kim et al., 2003). Inactive integrins are in a bent conformation with closely juxtaposed cytoplasmic tails, while active Integrin extend their extracellular domains and their cytoplasmic tails separate (Fig. 6C) (Hynes, 2002). Thus, FRET efficiency declines during integrin activation (Kim et al., 2003). Both the tissue surface of the PSM and the somite boundaries have an ECM of fibronectin and Fibrillin 2b (Fig. 6B). For technical reasons, we performed FRET-FLIM measurements along the somite boundaries. In agreement with a prior study that used a different protocol for measuring FRET-FLIM, we found increased integrin activation on the somite border relative to the tissue mesenchyme (Fig. 6D,E) (Sun et al., 2021). This increased activation was lost in *fn1a^−/−^; fn1b^−/−^* embryos, indicating that there is no fibronectin-independent activation of Integrin α5β1 specific to the boundaries of the paraxial mesoderm.

## DISCUSSION

Altogether, our data reveal a tissue fluidity code in which solidification is promoted by Cadherin 2 in parallel with Integrin α5 and fibronectin, whereas negative feedback by Fibrillin 2b promotes fluidization (Fig. 6F). Our data suggest that the cell speed of PSM cells declines as part of the differentiation process independently of jamming (Fig. 6F). This decline in cellular kinetic energy reduces the adhesion energy necessary for jamming/solidification to occur. A metabolic gradient has been linked to the decline in cell speed in the chick presomitic mesoderm (Oginuma et al., 2017). There are also changes in metabolism in the zebrafish tailbud (Ozbudak et al., 2010), thus the jamming-independent decline in cell speed may be due to metabolic changes during paraxial mesoderm differentiation.

In conjunction with the decline in cellular energy during PSM differentiation, we find that the adhesive energy of PSM cells increases. As cells differentiate from fluid mesodermal progenitors in the PZ to solidifying PSM, the cells stabilize Cadherin 2 in cell adhesions and assemble an ECM on the tissue surface, suggesting that the PSM is entering an adhesion-dominated regime (Fig. 6F). The increase in cell–cell and cell–ECM adhesion leads to a further decline in cell speed during solidification. Both *fibronectin 1a* and *fibronectin 1b* mRNA expression increases during this state transition, but *cadherin 2*, *integrin α5*, *integrin β1* and *fibrillin 2b* mRNAs do not (Genuth et al., 2023). Thus, the increase in stable Cadherin 2 in the PSM must solely be under post-transcriptional control.

The redundancy between *cadherin 2* and *integrin alpha 5/fibronectin* in PSM solidification is supported by *in vitro* studies. Cadherins drive cell aggregation by reducing interfacial tension between cells (Brodland, 2002). Similarly, Integrin α5β1-fibronectin adhesion can promote a liquid-to-solid transition in cell aggregates (Caicedo-Carvajal et al., 2010).

What underlies the solidifying effect of fibronectin and the fluidizing effect of Fibrillin 2b? Human dermal fibroblasts, which descend from the PSM, are migratory and have ruffled edges when plated on Fibrillin 1, whereas they are non-motile when spread on a fibronectin substrate (Bax et al., 2003). While both fibronectin and fibrillin proteins have RGD sequences, Integrin α5β1 also binds to a second ‘synergy site’ in fibronectin. The synergy site is engaged under tension and forms a catch bond that is stronger than binding to RGD alone (Schwarzbauer and DeSimone, 2011). Thus, the Integrin α5β1-fibronectin bond may be under greater tension than the Integrin α5β1-Fibrillin 2b bond, which could lead to differences in mechanotransduction (Sun et al., 2016). Interestingly, other cellular systems also exhibit distinct fibronectin and fibrillin ECMs. Spheroids of either human mesenchymal stem cells or fibroblasts assemble a fibronectin matrix on the surface of the spheroid but deposit a fibrillin matrix throughout the spheroid (Godwin et al., 2019; Redondo-Castro et al., 2018). By contrast, 2D fibroblast cultures create a single ECM composed of both fibrillin and fibronectin (Godwin et al., 2019).

*In vitro*, fibronectin is required for cells to assemble other ECM fibers including Fibrillin 1, type I collagen, perlecan, decorin and biglycan (Dallas et al., 2006). We find that fibronectin is required for Fibrillin 2b matrix assembly in the paraxial mesoderm but not in other tissues, such as the notochord and lateral plate mesoderm. What could be the role of fibronectin-dependent assembly of Fibrillin 2b in the paraxial mesoderm? One possibility is that the fluidizing effect of Fibrillin 2b modulates shear stress between the paraxial mesoderm and the notochord and neural tube to facilitate relative movement of the tissues. These tissues are bound by fibronectin-mediated adhesion, and cross-tissue solidification would impair relative movement between tissues (Dray et al., 2013; Guillon et al., 2020). After somite formation, tissue shear between the paraxial and axial tissues is responsible for generating the chevron shape of the zebrafish myotome (Tlili et al., 2019). In addition, PSM-derived myofibers pull on the notochord ECM and transfer segmental information to the notochord to pattern vertebral centra in zebrafish (Wopat et al., 2024).

The most severely affected embryos, *cdh2^−/−^; fn1a^−/−^; fn1b^−/−^* and *cdh2^−/−^; MZitgα5^−/−^*, exhibit a medial to lateral difference in phenotype with the medial PSM having the stronger phenotype. The cause of this difference is not clear. The medial PSM cells include the adaxial cells, which are induced by hedgehog signaling from the notochord and have a columnar morphology that is distinct from the other PSM cells (van Eeden et al., 1996). The adaxial cells are also the first to initiate myogenesis (Devoto et al., 1996; Felsenfeld et al., 1991). These differences may make the medial cells more sensitive to the loss of cell–cell and cell–ECM adhesion.

Another notable difference is the stronger phenotype of *cdh2^−/−^; MZitgα5^−/−^* relative to *cdh2^−/−^; fn1a^−/−^; fn1b^−/−^*. Genetically, this indicates that *integrin alpha 5* has a function independent of fibronectin genes. Thus, we sought a second *integrin alpha 5* ligand and analyzed *fibrillin 2b*. Surprisingly, loss of *fibrillin 2b* made the *cdh2^MO^; fn1a^−/−^; fn1b^−/−^* phenotype even less severe, which revealed the opposing functions of fibronectin genes and *fibrillin 2b* in PSM morphogenesis. The fact that concomitant loss of both *cadherin 2* and *integrin alpha 5* is more severe than concomitant loss of both *cadherin 2* and the three ECM proteins indicates that *integrin alpha 5* has a function independent of the fibronectin genes and *fibrillin 2b*. The FRET analysis in Fig. 6 does not suggest the presence of another *integrin alpha 5* ligand in the PSM. Thus, perhaps *integrin alpha 5* has a ligand-independent function in the PSM. In this regard, we note again the association of Integrin α5 on adjacent PSM cells along with Cadherin 2 (Jülich et al., 2015). A ligand binding-deficient mutant of Integrin α5 retains this association, and perhaps this association mediates weak adhesion that promotes PSM solidification independently of Integrin α5 ligand.

Cadherin 2 and Integrin α5-fibronectin promote PSM solidification in parallel by operating in the central tissue mesenchyme and tissue surface, respectively. This is a heterotypic redundancy involving different molecular processes, i.e. cell–cell adhesion and cell–ECM adhesion. It is also heterotopic redundancy involving different cells in different regions of the tissue. Cell–cell adhesion and cell–ECM adhesion also work in opposition. Cadherin 2 represses Integrin α5 activation in the central PSM mesenchyme, and the neural tube closure defect of *cdh2^−/−^* embryos is rescued by loss of Integrin α5 (Guillon et al., 2020; Jülich et al., 2015). Different cadherins drive cell sorting within the neural tube via differential adhesion (Tsai et al., 2020). Similarly, we find that cell–ECM adhesion can promote either tissue solidification via fibronectin or tissue fluidization via Fibrillin 2b. Thus, cell–cell adhesion and cell–ECM adhesion interact along many dimensions in the emergence of tissue and inter-tissue organization of the vertebrate embryo.

## MATERIALS AND METHODS

### Animal husbandry

Zebrafish, *Danio rerio*, were housed and maintained according to standard protocols (Nüsslein-Volhard and Dahm, 2002) approved by the Institutional Animal Care and Use Committee at Yale University.

### Zebrafish strains

The TL strain was used as the wild-type line. Mutant alleles were: *cdh2^tm101^*, *itgα5th^l030^*, *fn1a^ya13Tg^*, *fn1b^ya14Tg^* and *fbn2b^ya17Tg^* (Guillon et al., 2020; Jiang et al., 1996; Jülich et al., 2005). *fn1a^ya15Tg^* served as the Fibronectin 1a-mNeonGreen knock-in allele (Jülich and Holley, 2024).

### mRNA synthesis and injections

Three micrograms of each of *pCS2+ bm40-mNG* (a kind gift from Sandrine Bretaud at the Institut de Genomique Fonctionnelle de Lyon, France), *pCS2+ mem-mRFP*, *pCS2+ H2A-mRFP*, *Integrin α5-Aquamarine*, *Integrin α5-mCitrine*, *Integrin β1a-mCitrine*, *Integrin β1a* and *Integrin α5-Aquamarine-mCitrine* fusion (Sun et al., 2021) were linearized with NotI-HF for 3 h at 37°C, followed by purification with the NEB Monarch DNA clean-up kit (NEB, T1030S). The transcription reaction was assembled with an mMESSAGE mMACHINE SP6 (Ambion, AM1340) and incubated for 2 h at 37°C. The reaction was cleaned via SpinColumns (Bio-Rad, 732-6250) and stored at −80°C.

Embryos for the tissue compaction analysis were co-injected with 300 ng/µl *bm40-mNG* and 150 ng/µl *mem-mRFP* mRNA, and embryos for the cell motion analysis were injected with 120 ng/µl *H2A-mRFP* mRNA, all at the one-cell stage. Where applicable, 800 µM *cdh2* morpholino (5′-tctgtataaagaaaccgatagagct-3′) was added to the respective mRNA. Injected embryos were incubated at 28.6°C for 4 h, then cooled to 23.6°C for 3 h and further cooled to 22°C to slow down the speed of development.

### Imaging

Starting at the 10- to 12-somite stage, 3D timelapses of *H2A-mRFP* mRNA-injected embryos were acquired on a Zeiss LSM 880 fitted with a Linkam Scientific PE100 stage to ensure a constant temperature of 18°C.

Confocal *z*-stacks of *bm40-mNG; mem-mRFP* mRNA-injected embryos and embryos expressing *fbn2b-mScarletI* and/*or fn1a-mNG* for ECM fiber analysis were acquired on a Zeiss LSM 880 Airyscan NLO with the C Apo 40×/1.2 W objective at the 10- to 12-somite stage.

Images and tile scans comparing Fbn2b-mScarletI and Fn1a-mNG localization were taken on a Zeiss LSM 880 Airyscan NLO equipped with EC PlnN 10×/0.3, PlnApo 20×/0.8 and C Apo 40×/1.2 W objectives.

All embryos were embedded in 1.2% low-melt agarose (American Bioanalytical, AB00981; dissolved in E2). Embryos older than 24 hours post-fertilization were immobilized with 200 mg/ml Tricaine-S (Syndel/Western) added to the E2.

Airyscan images were processed in ZEN 2.3 SP1 with standard parameters provided by Zeiss.

Brightfield images of embryos embedded in 3% methylcellulose were captured on an Olympus MVX10 using its cellSens Standard software.

Images were cropped in ImageJ/Fiji and adjusted for brightness or contrast. 3D confocal projections were processed in Imaris (Oxford Instruments).

### Generation of *fibrillin2b-mScarletI* knock-in

The insertion site for the sequence encoding mScarletI (Eurofins gene synthesis and codon-optimized) into the endogenous locus of *fibrillin2b* was chosen based on Jensen et al. (2014) and lies within a flexible and unstructured region near the N terminus of the peptide, just downstream of a Furin-cleavage site, between Gly48 and Gln49.

To facilitate CRISPR/Cas9 mediated gene-editing gRNA target sites were identified using CHOPCHOP (https://chopchop.cbu.uib.no/) and CRISPOR (http://crispor.tefor.net/). gRNA target sequences closest to the insertion site were tested for cutting efficiency, opting for the target site 5′-cttaccccctgagggactcc*tgg*-3′, 5 bp 3′ to the insertion site. The guide DNA plasmid was generated as described by Hwang et al. (2013) and the gRNA synthesized with HiScribe T7 according to the manufacturer's instruction (NEB, E2040S).

To generate the donor plasmid, approximately 150 bp of genomic sequence flanking the insertion site within exon 1 of *fbn2b* were amplified from Tü and TL genomic DNA via PCR and verified by sequencing. A gene cassette composed of these homology arms, linker, the sequence encoding mScarletI and flanked by the gRNA target sequence on either end was then synthesized by Blue Heron/Eurofins. Furthermore, silent mutations were introduced into the gRNA target sequence within the cassette to prevent cutting: 5′-cttacCCtCTcAGGctCTCCTG-3′. This cassette was ligated into the pKHR4 plasmid (a gift from David Grunwald; Hoshijima et al., 2016). On the day of injections, 335 ng/µl Cas9 protein (PNA Bio, CP01) was gently mixed with ∼150 ng/µl gRNA and incubated at 37°C for 10 min to allow complex formation, then stored on ice before adding 50 ng/µl donor plasmid and Phenol Red. The mixture was injected into TL embryos at the one-cell stage within 20 min of fertilization. Injected embryos were incubated at 28°C, sorted for fluorescence at 24 hours post-fertilization and raised to adulthood. Founder fish were identified by crossing to wild type, and fluorescent F1 embryos were raised to establish stable lines.

### Generation of *fibrillin 2b* mutant alleles

*fbn2b* mutant alleles were generated using CRISPR/Cas9 and a Stop codon cassette DNA repair template (Gagnon et al., 2014) targeting sites in exon 2 and exon 3. Guide DNA templates and gRNA were synthesized as described by Gagnon et al. (2014). The exon 2 target site sequence 5′-gatacttacgaactatacac*tgg*-3′ and exon 3 target site sequence 5′-gcaatctgtaggaactcctg*tgg*-3′ were identified using CHOPCHOP (https://chopchop.cbu.uib.no/). gRNA, Cas9 mRNA and respective Stop codon oligos were injected into TL embryos at the one-cell stage (exon 2 Stop oligo: 5′-cttcctggagggaaccagtgctacaacagcttaattaaggtttaaacgccatgactatagttcgtaagtatcccc-3′; exon 3 Stop oligo: 5′-agcaaaagccatcaccacagctacaacagcttaattaaggtttaaacgccatgacgagttcctacagattgcttgg-3′). At the 10- to 12-somite stage genomic DNA was extracted from 12 injected embryos and the insertion verified by PCR (exon 2: *fbn2b_ex2_diagA* 5′-ccaacgtatgcggttcccgcttc-3′; *STOP_A* 5′-gcttaattaaggtttaaacgcc-3′; exon 3: *fbn2b_ex3_diagA* 5′-ttcatctacctgtttggctttg-3′; *STOP_A* 5′- gcttaattaaggtttaaacgcc-3′). Injected embryos were raised to adulthood and crossed to wild type to identify founder fish.

### Microscopy quantification

For analyses of cell shape and packing fraction, embryos expressing membrane RFP and secreted GFP were used. For packing fraction quantification, the GFP channel was preprocessed by manually subtracting the background, and then scaling the brightness using the membrane channel as a standard candle. Specifically, we used the 95th percentile pixel in each *z*-slice as the comparator. Images were thresholded in ImageJ by Phansalkar's algorithm with a radius of 15. Occasional intracellular vesicles were manually removed from the binary image. Further analysis was performed using MATLAB R2023a. The PSM area was defined for each transverse slice as the convex hull around the GFP-negative region, excluding any intrusions of other tissues. The packing fraction was calculated as the percentage of GFP-negative pixels within the PSM volume. The packing fraction distribution was estimated by randomly sampling 100,000 20 µm by 20 µm boxes in the transverse orientation that at least partially overlapped the PSM. A weighted kernel density estimate was calculated with the ‘ksdensity’ function in MATLAB. Statistics were calculated by unpaired, two-way, two-tailed Student's *t*-test with Hommel's method to control the family-wise error rate in R.

For cell shape analysis, PSM cells were segmented using Cellpose 2.0 (Pachitariu and Stringer, 2022). Custom models were trained for the coronal and transverse orientations using wild type, *cdh2^−/−^* and *MZitgα5^−/−^; cdh2^−/−^* embryos. The fine-tuned Cellpose 2.0 models are available on Dryad at doi:10.5061/dryad.wstqjq30g (Genuth et al., 2025). In the coronal orientation segmentation of the two-color images was performed using the grayscale option in Cellpose. For the transverse orientation, we used a custom weighting of the channels by adding a quarter of the secreted-GFP intensity to the membrane channel. The performance of the models was audited by spot checking images from each genotype. Slices every 5 µm were analyzed. Circularity was calculated using the ‘regionprops’ function in MATLAB:

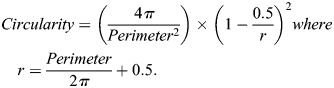


### ECM quantification

To analyze the ECM structure, a maximum projection of the dorsal surface of the PSM was created. mRNA encoding membrane GFP was injected into *fn1a^−/−^; fn1b^−/−^* embryos to aid anatomical identification. Regions of interest (ROIs) were created by manually tracing the tissue boundary. Quantifications were carried out using MATLAB. To calculate the packing fraction, ECM brightness was scaled from 0 to 1 and the fibers were enhanced using the ‘fibermetric’ function in MATLAB. Images were then automatically thresholded using Otsu's method. ECM packing fraction was defined as the percentage of positive pixels within the ROI. The packing fraction variance was calculated using a 10 µm^2^ sliding window.

Analyses of the nematic order of the fiber orientation were performed as described by Maroudas-Sacks et al. (2021). Briefly, the nematic director of each pixel was calculated using the averaged squared gradient in a given window (Bazen and Sahib, 2000). A Gaussian weighted average with a standard deviation of 5 pixels was used. Coherence was defined as 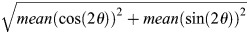 in a 10 µm by 10 µm window. Coherence of 0.75 was used as the cutoff to define regions of ordered fibers. Statistics were calculated using unpaired, two-way, two-tailed Student's *t*-test with Hommel's method to control the family-wise error rate in R.

### Cell motion analysis

Embryos were mounted in 1% low-melt agarose in E2 and imaged every 3 min for 90 min at 18°C. Cells were tracked using the Brownian spot tracker algorithm in Imaris with no gaps allowed. The average motion of the anterior-most 50 µm of the PSM was used as the frame of reference. The mean and standard deviation of the PSM cell velocities were calculated for each embryo.

To compare cell speeds between the wild-type PZ and PSM, we quantified cell speed from four embryos from Lawton et al. (2013) and three from Dray et al. (2013). Velocity is defined as: 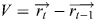 where 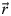 is the cell's *xyz* coordinate. To account for embryo-to-embryo variability, we subtracted the PSM speed from the PZ speed in each embryo and then averaged. We compared this average difference in speed between PZ and PSM to the average difference in speed between PSM cells with fewer than two neighbors and PSM cells from the bulk population in three *cdh2^−/−^; fn1a^−/−^; fn1b^−/−^* embryos and four *cdh2^−/−^; MZitgα5^−/−^* embryos (Fig. 3C) by unpaired, two-way, two-tailed Student's *t*-test.

Cell tracks were used to calculate the number of neighbor exchanges within a given section of tissue over a fixed 45-min window. Nucleus positions were preprocessed by filtering out any nucleus tracks that were not detected in each frame of the 45-min window. Delaunay triangulations were performed on the filtered positions for each frame to determine nearest neighbor cells. Cells were considered neighbors if they shared a Delaunay edge of at most 12 µm, slightly larger than a cell diameter. The distance criterion was calibrated to ensure that cells had at most 12 neighbors in 3D. This same distance cutoff was used to identify isolated cells with fewer than two neighbors for speed comparisons.

### The deformable particle model

To model cell behavior in the PSM, we conducted discrete element method (DEM) simulations of 2D deformable particles that represent cells. Each cell i obeys the following shape-energy function:

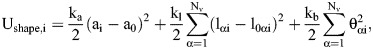
where each cell is represented by a set of N_v_ vertices labeled by α that form a polygon (see Fig. 4A). The bonds between vertices are the vectors 
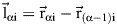
, with vertex positions 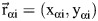, cell rest area a_0_, and membrane rest length l_0αi_. The cell area stiffness k_a_ and segment length stiffness k_l_ penalize changes in the cell area a_i_ and membrane lengths l_αi_ from their rest values. The circularity C is defined as the ratio of the cell area and squared perimeter. 
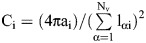
 for cell i ranges from 0 to 1, with a circle having C_i_=1. The cell membrane bending stiffness k_b_ sets the energy cost of membrane curvature, which depends on the angle θ_αi_ between 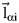 and 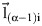. For a full list of parameters used in the DEM simulations, refer to Table S1.

We assumed that the interaction energy U_int_ between cells is defined by a pair potential that is a function of the distance d_αi,βj_ between the membranes of two cells (vertex αi and line segment βj):
(1)

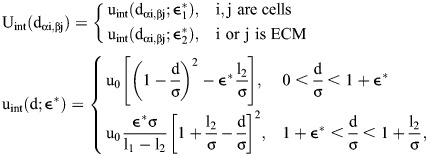
where u_0_ is the cell membrane interaction energy, 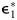 is the cell-cell adhesion strength, 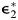 is the cell-ECM adhesion strength, σ sets the repulsive interaction lengthscale, l_2_=0.3σ is the adhesion range of the cell membrane (see Fig. 4A, Fig. S6B,C). Quantities indicated with an asterisk have been made dimensionless. In Table S1, 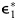 and 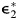 refer to independent values of 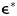 used for cell–cell and cell–boundary adhesion, respectively. The equation for the distance between membranes has no component tangential to the membrane to give smooth sliding intercellular forces (Ton et al., 2024). The total potential energy U incorporates single-cell shape energy, cell–cell adhesion, and cell–ECM adhesion:
(2)

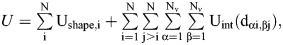
where N is the number of cells. The surface-bound fibronectin is modeled as a large deformable particle that surrounds the cells. The deformable particle (DP) stiffness parameters for the fibronectin boundary are given in Table S1.

The activity of a cell i is modeled after an active Brownian process with a force given by:

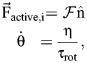
where 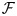 is a random number sampled uniformly from the range [0, 2f_0_*] at each timestep, f_0_ sets the cell activity force scale, 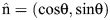, η is a Gaussian random number in the range [0,1], and τ_rot_ is the timescale of the rotational diffusion (see Fig. S6A).

### Simulation protocol

To generate initial states for PSM transverse sections, we first placed cells randomly within a circular boundary with an initial packing fraction of *φ*=0.7, then compressed the system in packing fraction increments of Δ*φ*=0.005 until *φ*=0.8. The packing of cells was achieved by compressing the soft boundary around the cells in small steps, with each step followed by energy minimization. During this quasistatic compression, the FIRE algorithm (Bitzek et al., 2006) was used to minimize the total potential energy 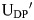:

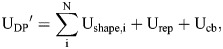
where U_rep_ is equal to U_int_ with 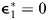 and:

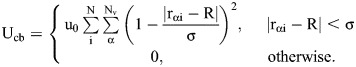
Here, r_αi_ is the distance between a vertex α on cell i and the center of the circular boundary of radius R. u_0_ sets the strength of the interaction energy between the circular boundary and the vertices, and is chosen to be the same as the membrane interaction energy strength in Eqn 1.

The soft ring boundary was then tiled with vertices and converted to a DP to represent the surface-bound fibronectin. We then equilibrated the simulations by running overdamped dynamics (Eqn 3) with cell activity and the full DP potential energy (Eqn 2) including cell–cell and cell–ECM adhesions.

Each vertex α on cell i obeys the overdamped equation of motion:
(3)

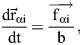
where 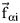 is the total force experienced by vertex i and b is the damping coefficient. An overdamped equation of motion is commonly used to remove the energy input due to cell activity. We integrated Eqn 3 using the Euler method with timestep dt*=0.001, as is typically done in the overdamped limit (Barton et al., 2017; Erdemci-Tandogan and Manning, 2021; Kursawe et al., 2017; Rozman et al., 2023).

To calculate neighbor exchange rates, we took configurations from the simulations at regular intervals of 5τ_rot_* (2.5 min). We then used the FIRE algorithm with a force tolerance of 0.01f_adh_* (0.07 nN) without cell activity to find a force balanced configuration. Two cells were defined as neighbors if they were in contact, and we counted neighbor exchanges as changes in neighbors from one energy minimized configuration to the next.

### Modeling repression of Integrin α5 activation in the tissue mesenchyme by Cadherin 2

In addition to the potential energy in Eqn 2, we conducted simulations (red data points in Fig. 4C) in which we incorporated the effects of cadherin suppressing expression of integrin. To do this, we introduced an additional energy term, 
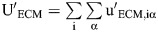
, where:

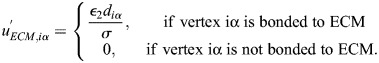
Here, 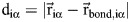 is the distance between vertex α on cell i and a position 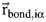 to which the vertex is bonded. The integrin-fibronectin bonds have an association rate k_on_ and disassociation rate k_off_. At time t, the bond exists between r_iα_(t) and 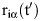, which is the vertex position at the time 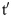 of bond association. This model is motivated by the presence of ECM within the tissue wherever cadherin–cadherin bonds are not present. The bond between the membrane at vertex iα and the ECM has a characteristic lifetime determined by k_off_, but the bond also breaks and will not reform as long as vertex iα is involved in a cell–cell adhesion.

### Conversion between dimensionless numbers and physical quantities

To convert from simulation units to physical units, we used three physical quantities to set the mass, length and time scales (see Table S1). The three quantities we employed are the typical PSM cell cross-sectional area a_0_, estimated cohesion between zebrafish embryonic cells f_adh_, and the rotational diffusion timescale τ_rot_. We were able to estimate a_0_ from our imaging experiments, f_adh_ from atomic force microscopy measurements (Kashef and Franz, 2015; Krieg et al., 2008) and τ_rot_ from track straightness measurements in the zebrafish PSM. Using these three quantities, we were able to fully specify the physical dimensions of all quantities in the simulation. For example, we the dimensionless velocity of a cell can be converted to μm/minute. The dimensionless velocity is given in units of length 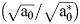 over time (τ_rot_/τ_rot_*), so a cell velocity of v*=0.01 corresponds to 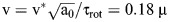
m/minute. Likewise, dimensionless forces are given in units of f_adh_/f*_adh_. The cell activity force scale f*_0_=0.1 corresponds to f_0_=f_0_*(f_adh_/f*_adh_)=0.71 nN.

We estimated the unsticking force by 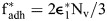 (Ton et al., 2024). The factor of N_v_/6 came from the assumption that cells have one-sixth of their membrane in contact with an adjacent cell on average. The maximum dimensionless adhesion force in Eqn 1 is 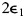. We used the wild-type value of adhesion (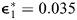) to set the adhesion force scale, found by a simulation parameter sweep in 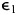. From atomic force microscopy measurements, an estimate of homotypic cohesion between zebrafish mesodermal cells is around ∼5 nN (Krieg et al., 2008). Our predicted Cadherin 2 mutant value of adhesion was 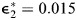, which corresponds to 2.1 nN. This prediction agrees with the range reported for zebrafish mesodermal cells grown from E-cadherin morpholino-expressing progenitors (Krieg et al., 2008).

### FRET-FLIM

FLIM was performed on a Leica STELLARIS 8 confocal microscope with a 40× HC PL APO water immersion objective with a 1.1 numerical aperture and a pinhole size of 1.7 airy units (131.2 µm). The donor was excited using a white light laser set to 440 nm. Donor florescence was collected in the 450-500 nm range using a HyD X detector. Reflected laser light was blocked with the 448/514/561 notch filter. Samples were scanned with a scan speed of 400 Hz, a repetition rate of 80 MHz and a line accumulation of 16. The pixel dwell time was 3.1625 µs and the pixel size was 2.27 µm. A time-correlated single photon counting (TCSPC) system was used to record photon events. Data acquisition and analysis were performed using the Leica Application Suite X software with the LAS X FLIM/FCS module. Leica's high-speed FLIM filter was used to analyze only single-photon events. For each sample, pixels were binned into either a single mesenchymal cell (MC) or somite boundary cell (SB) ROI. For each ROI, two-exponential reconvolution fitting was used to fit fluorescence decays and samples with a peak maximum over 1000 for both ROIs were kept for analysis. Donor-only lifetime (*τ*_*D*_) was determined using Integrin α5-Aqm co-injected with unlabeled Integrin β1-mCit. ROIs were drawn for the MC and SB and then the amplitude weighted average lifetime (*τ*_*AvAmp*_) was calculated for seven samples and averaged to obtain*τ*_*D*_. This donor-only lifetime was used to calculate FRET efficiency (*E*) using two components (*n*) with the following formula:

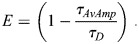
*τ*_*AvAmp*_ is the mean decay time (amplitude weighted average lifetime), where *A* are the amplitudes, *τ* are the exponential decay times, the index *k* indicates a sum over exponential components, and *n* is the total number of exponential components:

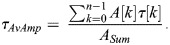
*A*_*Sum*_ is the sum of fluorescence intensity for all components at time zero:

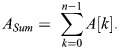
FLIM-FRET data were analyzed using GraphPad Prism with one-way ANOVA with Bonferroni correction for multiple comparisons.

## Supplementary Material

10.1242/develop.204874_sup1 Supplementary information
