# Supplementary material for: A cadherin-integrin–ECM code for presomitic mesoderm fluidity
Source: Development. 2025 Nov 3;152(21):dev204874. doi: 10.1242/dev.204874 (PMC12633790; doi:10.1242/dev.204874)
Supplement: Supplementary information [file develop-152-204874-s1.pdf]

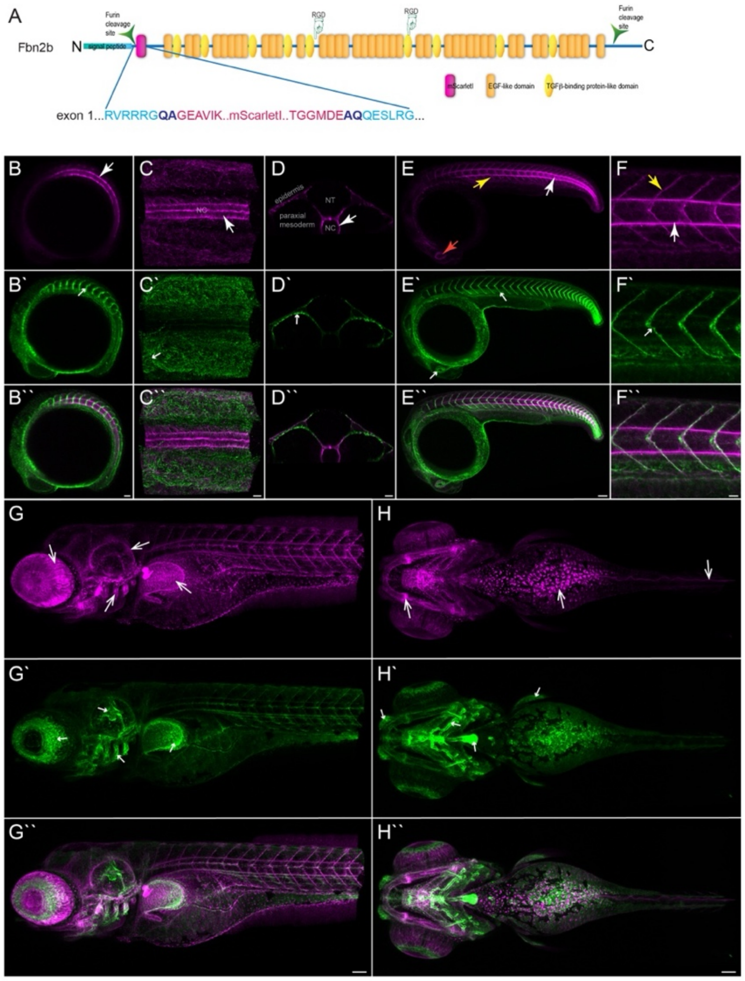

Fig. S1. Endogenous Localization of Fbn2b-mScarletI. (A) Schematic of the domain structure of Fbn2b and insertion site for the fluorescent protein mScarletI. Indicated are the Furin cleavage sites and the RGD domains. The peptide sequence at the insertion site is detailed: mScarletI in magenta, the linker sequences are in dark purple and the Fbn2b sequence is in light blue. The knock-in cassette was designed such that the fluorophore is flanked by structurally neutral linker residues Gln and Ala on its N-terminus and by Ala and Gln C-terminally. These linkers replace the first 4 amino acids (MVSK) and last 3 amino acids (LYK) of mScarletI. Crosses between *fbn2b*$^{+/-}$ and *fbn2b-mScarletI* resulted in complementation indicating the knock-in allele is functional (3 experiments, n=238, 0% of embryos positive for Fbn2b-mScarletI exhibited the *fbn2b*$^{-/-}$ phenotype). **(B-H")** 3D projections of confocal z-stacks showing localization of (B-H) Fbn2b-mScarletI and (B'-H') Fn1a-mNeonGreen at three developmental stages in live embryos. (B"-H") Overlay. (B-B") Lateral view of an embryo at the 10-12 somite stage. (B) White arrow localization of Fbn2b-mScarletI at the notochord. (B') White arrow Fn1a-mNeonGreen along the somite boundaries. (C-C") Dorsal view of the anterior PSM at the 10-12 somite stage. (C) White

Development • Supplementary information

arrow points to the notochord. (C') Fn1a-mNeonGreen forms a dense matrix atop the PSM. White arrow: somite boundary. (D-D") Transverse section through the anterior PSM of the same embryo as in (C-C''). (D) Fbn2b-mScarletI deposition along the epidermis, and it ensheathes the notochord (white arrow). (D') Fn1a-mNeonGreen accumulation on the surface of the PSM. White arrow: dorsal surface of the PSM. (E-E") Lateral view at 24 hpf. (E, F) White arrow: notochord. Yellow arrow: myotome boundary. Red arrow: eye. (E', F') White arrow: Fn1a-mNeonGreen accumulation along the myotome boundary. Blue arrow: eye. (G-G") Lateral view of the anterior half of a 5 day old larvae. (G) White arrow: otic vesicle. Orange arrow: pectoral fin. Chartreuse arrow: branchial arches. Green arrow: eye. (G') White arrow: matrix spanning the lumen of the ear. Yellow arrow: branchial arches. Cyan arrow: retina. Red arrow: pectoral fin. (H-H'') Ventral view of the anterior part of a 5 day old larvae. (H) White arrow: jaw. Green arrow: epidermis covering the yolk. (H') Yellow arrow: branchial arches. White arrow: heart ventricle. Red arrow: pectoral fin. Magenta arrow: olfactory pits. (B-C", E-H'') Anterior to the left. NT notochord. NC neural tube. Scale bars: (B'') 50μm (C'', D'') 15μm (E'') 80μm (F'') 20μm (G'', H'') 80μm.

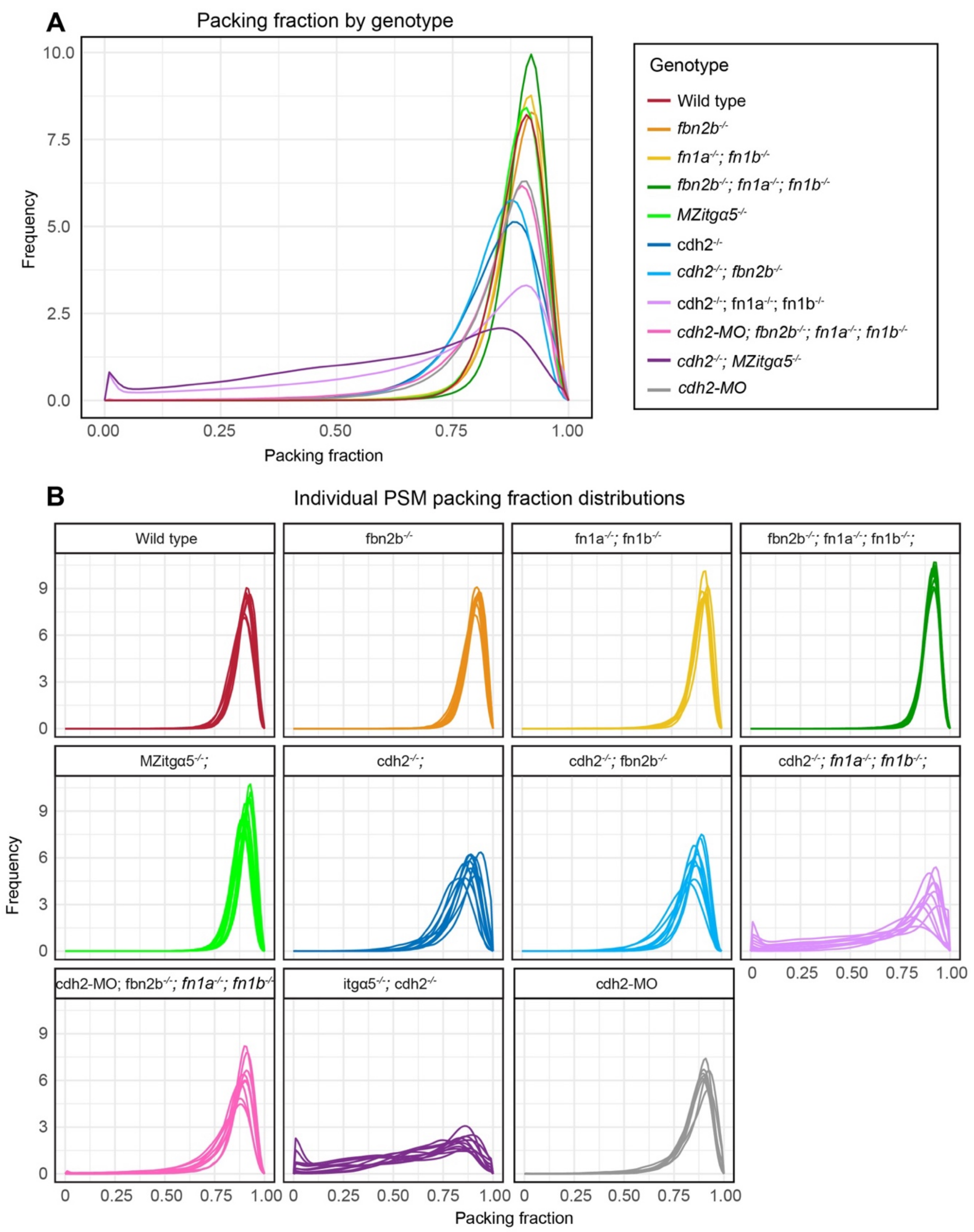

Fig. S2. Packing Fraction Distributions. Measured in randomly selected 20 x 20 μm windows and grouped by genotype (**A**) or individual PSM (**B**). Note that *cdh2*$^{-/-}$ (light blue) is very similar to *cdh2*-MO (grey). *cdh2*-MO PSMs have a mean packing fraction of 0.85, which is significantly lower than wild type ($p<0.001$) but statistically, indistinguishable from *cdh2*$^{-/-}$ embryos ($p=0.2$). Statistics calculated by Mann-Whitney U.

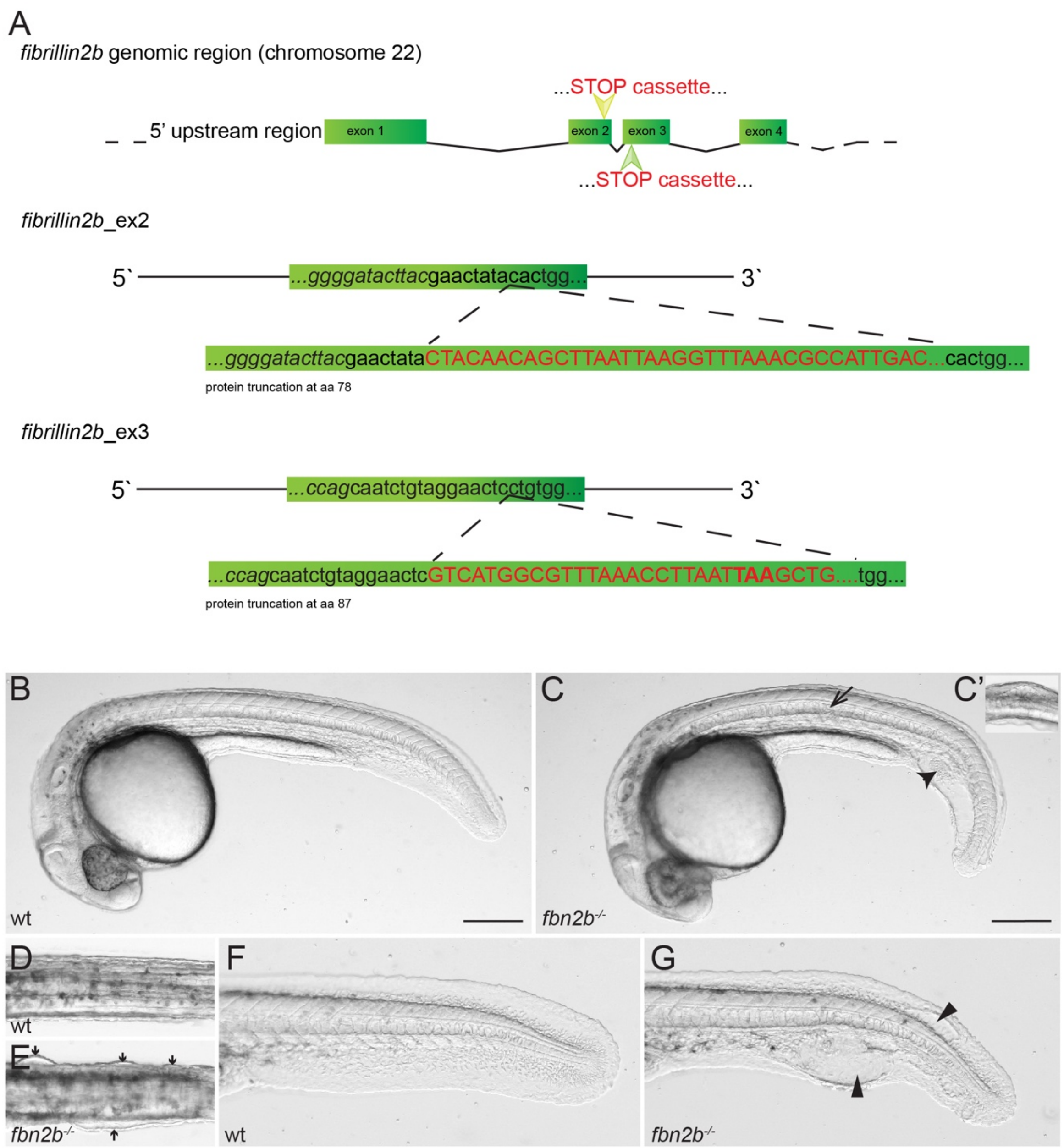

Fig. S3. Generation of *fibrillin 2b* Mutants.

(A) Schematic of the genomic locus of *fbn2b* on chromosome 22. *fbn2b* mutant alleles were generated using CRISPR/Cas9 and a Stop codon cassette targeting exon 2 and exon 3. The DNA sequence for the target site is in black with intron sequences in *italic* and the inserted stop codon cassette sequence in red. The two alleles do not complement the phenotype indicating the observed malformations are specifics to the *fbn2b* loss of function (3 experiments, n=213, 26.7% displayed the phenotypes described in (B-G)). **(B-G)** Brightfield images of wild type and *fbn2b* homozygous mutants. *fbn2b* homozygous mutants develop bends in the notochord (black arrow in C, magnified in C' insert) and a cavernous caudal vein with red blood cells accumulating within the edema (black arrowheads in C and G). These alleles also display skin distension over the trunk above the yolk extension previously reported for the *pfd*^{gw1} allele (black arrows in E)(Gansner*, et al*, 2008). (B, C, F, G) Lateral view with anterior to the left. (C', D, E) Dorsal view with anterior to the left. (B-C) 26 hpf and (D-G) 30 hpf. Scale bars 200μm.

Development • Supplementary information

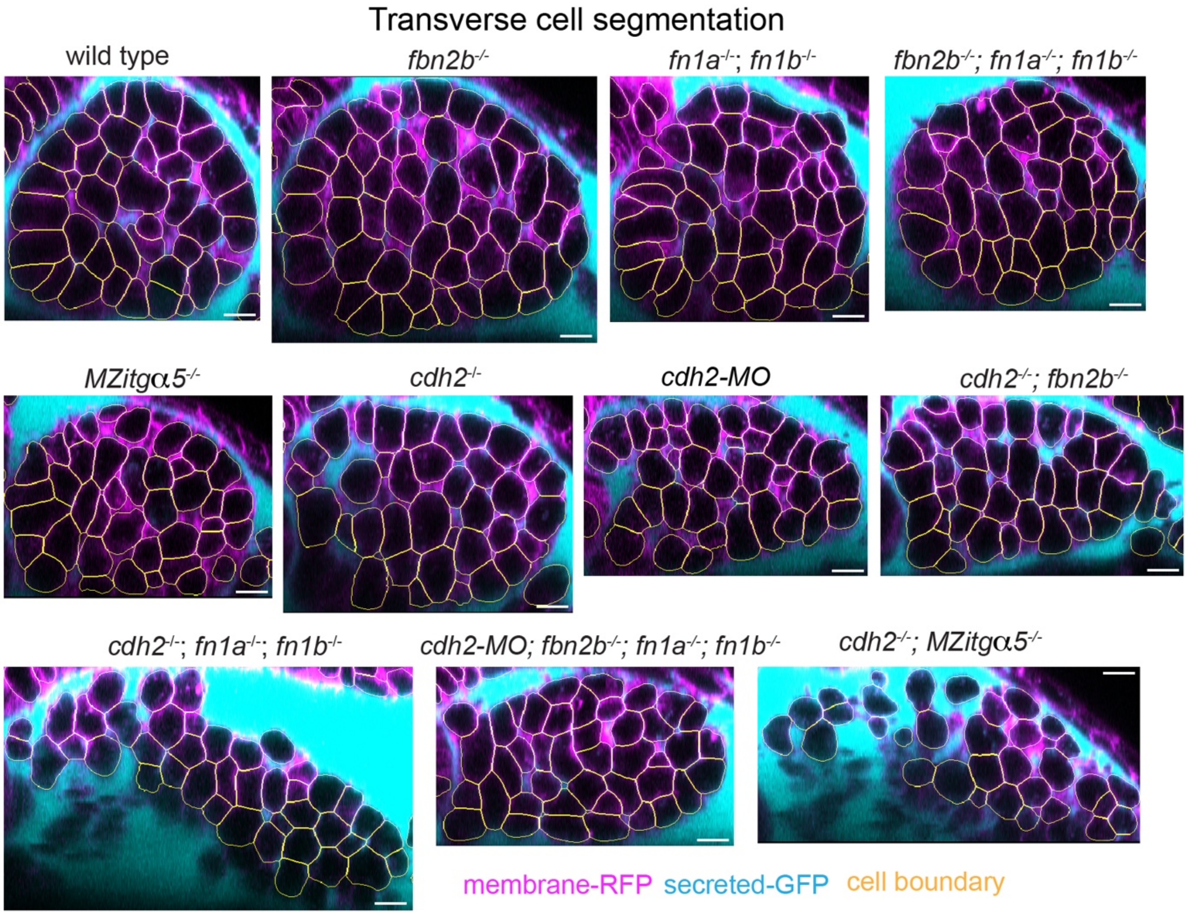

Fig. S4. Cell Shape Segmentation. Machine learning segmentation of PSM cell shapes. Scale bar =10 μm.

Development • Supplementary information

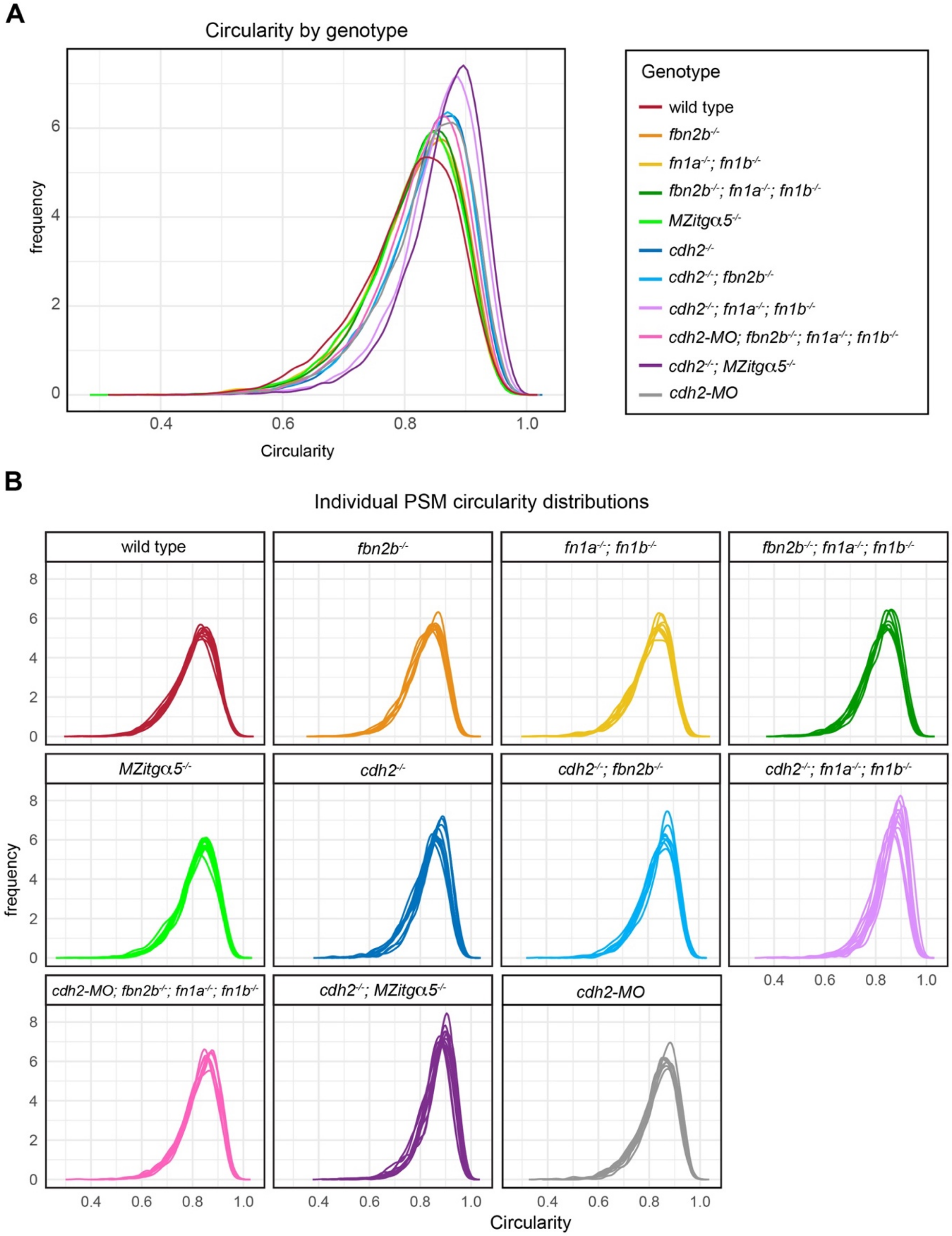

Fig. S5. Cell Shape Distributions. Distribution of cell shapes grouped by genotype (**A**) or individual embryos (**B**). *cdh2*-MO PSMs are more circular than wild type (mean=.83, $p<0.001$) but are statistically, indistinguishable from *cdh2*$^{-/-}$ PSMs ($p=0.96$). Statistics calculated by Mann-Whitney U.

Development • Supplementary information

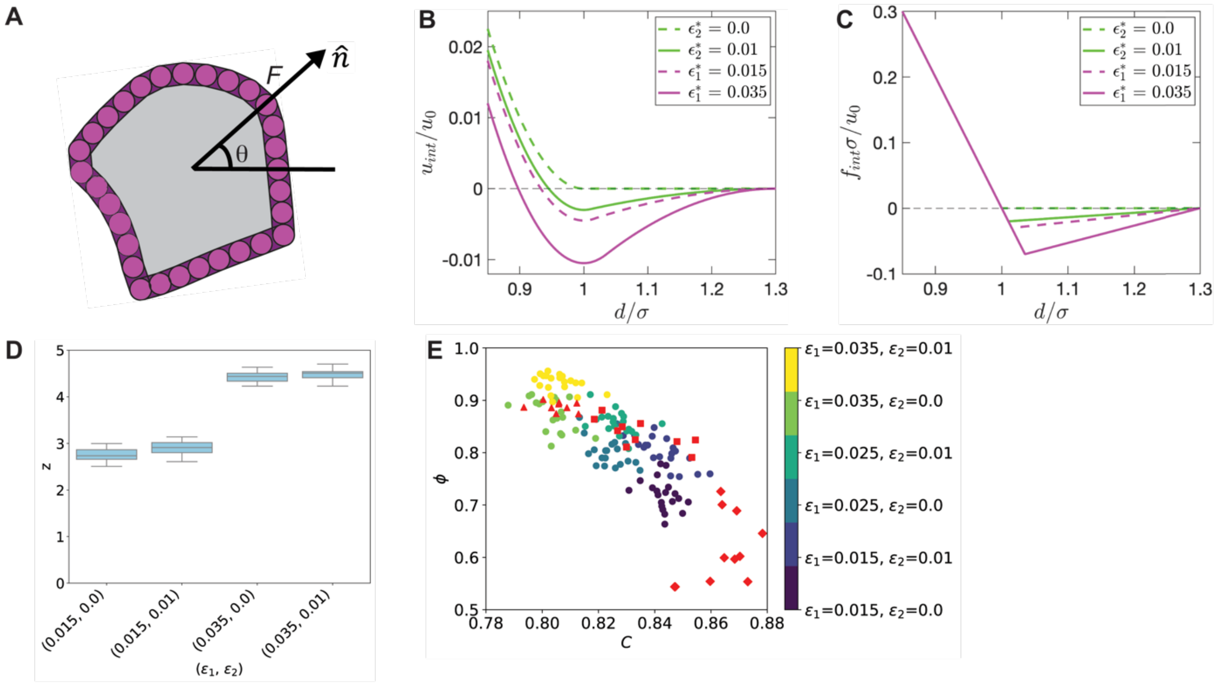

Fig. S6. Cell Activity, Cell-Cell Interaction, Cell Contact Number, and Packing Fraction-Circularity Relationship. (A) Cell activity consists of a driving force F along the direction $n\hat{}$, which has a diffusing angle θ. **(B)** The interaction potential u_{int} (in units of u_0) is plotted as a function of distance $^*_2$d (in units of σ) between a vertex and a line segment. **(C)** The corres$^*_1$ponding force f_{int} (in units of σ/u_0) from u_{int} is plotted as a function of distance d (in units of σ). Various values of ϵ^* are plotted, where ϵ (green) represents the mutant and wild-type values of cell-ECM adhesion, and ϵ (magenta) represents the mutant and wild-type values of cell-cell adhesion. **(D)** The number of cell contacts *z* is shown for values of ϵ_1 and ϵ_2. **(E)** Packing fraction ϕ is plotted against circularity C for various values of ϵ_1 and ϵ_2. Data from wild-type (red triangles), cadherin-2 mutant (red squares), and cadherin-2/integrin α5 mutant (red diamonds) embryos are also shown for comparison. For each parameter combination, simulations are performed for 20 random initial conditions.

Development • Supplementary information

Table S1. Simulation Parameters. Parameters and other quantities used in the DEM simulations. Asterisks denote dimensionless simulation units. Quantities in bold and italics (f_{adh}, a_0, τ_{rot}) have been converted into physical units based on experimental measurements. All other parameters are given physical units based on the bolded and italicized quantities. For the simulations in Fig. 4C, all parameters have been held constant other than $\epsilon_1$and ϵ_2.

Simulation parameters	Symbol	Value	Value (with units)
**Parameters used for U_{int}**			
Unsticking force	$\mathbf{f_{adh}}$	$\mathbf{2\varepsilon_1^* N_v/3 = 0.7}$	**5 nN**(Krieg, *et al*, 2008)
**Cell-cell adhesion strength (WT)**	ε_1^*	0.035	f_{adh} = 5 nN
**Cell-cell adhesion strength (cadherin mutants)**	ε_1^*	0.015	f_{adh} = 1.43 nN
**Cell-ECM adhesion strength (WT)**	ε_2^*	0.01	f_{adh} = 2.14 nN
**Cell-ECM adhesion strength (ECM mutants)**	ε_2^*	0	f_{adh} = 0 nN
**Interaction lengthscale**	σ^*	l_0	0.53 μm
**Membrane interaction energy**	u_0^*	1	4.43×10^1nN·μm
**Cell-ECM binding rate**	k_{on}^*	1	2 min
**Cell-ECM unbinding rate**	k_{off}^*	0.1	0.2 min
**Parameters used for U_{shape}**			
Cell rest area	$\mathbf{a_0}$	**1**	**25π μm²**
**Number of vertices per DP**	N_v	30	-
**Cell rest circularity**	C	1	-
**Membrane segment rest length**	l_0^*	$\frac{1}{N_v}\sqrt{\frac{4\pi a_0^*}{C}}$	1.06 μm
**Cell area stiffness**	k_a^*	2.5	6×10^{-2} μm/nN³
**Boundary area stiffness**	k_a^*	0.1	2.4×10^{-3} μm/nN³
**Segment length spring constant**	k_l^*	0.25	0.31 μm/nN
**Boundary segment length spring constant**	$k_{l,bd}^*$	0.25	0.31 μm/nN
**Membrane bending rigidity**	k_b^*	0.01	4.43×10^{-1} nN·μm
**Boundary bending rigidity**	$k_{b,bd}^*$	0.1	4.43 nN·μm
**Parameters used for F_{active}**			
Rotational diffusion time	$\boldsymbol{\tau_{rot}}$	**1**	**0.5 min**
**Cell activity force scale**	f_0^*	0.1	0.71 nN
Parameters used for integration			
**Numerical integration timestep**	dt^*	1×10^{-3}	5×10^{-4} min
**Damping coefficient**	b^*	1	1.02 kg/min

Development • Supplementary information

Table S2. Statistical analysis for all genotypes. Full statistics calculated by Student's t-test with Hommel's correction to control the family-wise error rate. Yellow $p<0.05$, orange $p<0.01$, red $p<0.001$ (A) Adjusted p-values for PSM Cell Packing Fraction. (B) Adjusted p-values for PSM Cell Circularity for coronal and transverse planes. (C) Adjusted p-values for PSM cell velocity.

A PSM Packing Fraction

	wild type	*fbn2b*$^{-/-}$	*fn1a*$^{-/-}$; *fn1b*$^{-/-}$	*fbn2b*$^{-/-}$; *fn1a*$^{-/-}$; *fn1b*$^{-/-}$	*MZitgα5*$^{-/-}$	*cdh2*$^{-/-}$	*cdh2*-MO	*cdh2*$^{-/-}$; *fbn2b*$^{-/-}$	*cdh2*$^{-/-}$; *fn1a*$^{-/-}$; *fn1b*$^{-/-}$	*cdh2*-MO; *fbn2b*$^{-/-}$; *fn1a*$^{-/-}$; *fn1b*$^{-/-}$	*cdh2*$^{-/-}$; *MZitgα5*$^{-/-}$
wild type	NA	0.01396	0.43824	0.13213	0.06655	0.00065	5.5E-05	8.3E-05	2.5E-06	0.00091	4.27E-10
fbn2b$^{-/-}$	0.01396	NA	0.56834	0.98009	0.96535	0.05236	0.01428	0.01396	6.7E-05	0.27091	2.09E-08
fn1a$^{-/-}$; *fn1b*$^{-/-}$	0.43824	0.56834	NA	0.98009	0.98009	0.0065	0.00108	0.00118	1.3E-05	0.01673	5.22E-09
fbn2b$^{-/-}$; *fn1a*$^{-/-}$; *fn1b*$^{-/-}$	0.13213	0.98009	0.98009	NA	0.98009	0.08314	0.04596	0.03654	0.00011	0.38565	9.75E-07
MZitgα5$^{-/-}$	0.06655	0.96535	0.98009	0.98009	NA	0.01104	0.00162	0.00209	3E-05	0.02567	6.04E-09
cdh2$^{-/-}$	0.00065	0.05236	0.0065	0.08314	0.01104	NA	0.98009	0.98009	0.04652	0.98009	7.14E-04
cdh2-MO	5.5E-05	0.01428	0.00108	0.04596	0.00162	0.98009	NA	0.98009	0.01544	0.98009	4.04E-05
cdh2$^{-/-}$; *fbn2b*$^{-/-}$	8.3E-05	0.01396	0.00118	0.03654	0.00209	0.98009	0.98009	NA	0.02316	0.73419	9.73E-05
cdh2$^{-/-}$; *fn1a*$^{-/-}$; *fn1b*$^{-/-}$	2.5E-06	6.7E-05	1.3E-05	0.00011	3E-05	0.04652	0.01544	0.02316	NA	0.0021	0.77641
cdh2-MO; *fbn2b*$^{-/-}$; *fn1a*$^{-/-}$; *fn1b*$^{-/-}$	0.00091	0.27091	0.01673	0.38565	0.02567	0.98009	0.98009	0.73419	0.0021	NA	6.42E-06
cdh2$^{-/-}$; *MZitgα5*$^{-/-}$	4.27E-10	2.09E-08	5.22E-09	9.75E-07	6.04E-09	7.14E-04	4.04E-05	9.73E-05	0.77641	6.42E-06	NA

B PSM Cell Circularity

Coronal Orientation

	wild type	*fbn2b*$^{-/-}$	*fn1a*$^{-/-}$; *fn1b*$^{-/-}$	*fbn2b*$^{-/-}$; *fn1a*$^{-/-}$; *fn1b*$^{-/-}$	*MZitgα5*$^{-/-}$	*cdh2*$^{-/-}$	*cdh2*-MO	*cdh2*$^{-/-}$; *fbn2b*$^{-/-}$	*cdh2*$^{-/-}$; *fn1a*$^{-/-}$; *fn1b*$^{-/-}$	*cdh2*-MO; *fbn2b*$^{-/-}$; *fn1a*$^{-/-}$; *fn1b*$^{-/-}$	*cdh2*$^{-/-}$; *MZitgα5*$^{-/-}$
wild type	NA	0.27604	0.79509	0.79509	0.18152	4.7E-06	0.00055	8.6E-06	1.1E-08	0.00367	1.90E-10
fbn2b$^{-/-}$	0.27604	NA	0.02956	0.01433	0.79509	5.5E-06	0.00106	2.1E-06	3.9E-08	0.03208	9.18E-11
fn1a$^{-/-}$; *fn1b*$^{-/-}$	0.79509	0.02956	NA	0.79509	0.01429	2.7E-07	1.8E-05	2.6E-07	2.2E-09	0.00345	1.39E-11
fbn2b$^{-/-}$; *fn1a*$^{-/-}$; *fn1b*$^{-/-}$	0.79509	0.01433	0.79509	NA	0.00823	1.9E-07	1.1E-05	1.9E-07	1.6E-09	0.00245	1.09E-11
MZitgα5$^{-/-}$	0.18152	0.79509	0.01429	0.00823	NA	1.5E-05	0.00076	3.3E-06	2.4E-07	0.0542	1.66E-09
cdh2$^{-/-}$	4.7E-06	5.5E-06	2.7E-07	1.9E-07	1.5E-05	NA	0.01347	0.79509	0.00185	0.4134	1.02E-06
cdh2-MO	0.00055	0.00106	1.8E-05	1.1E-05	0.00076	0.01347	NA	0.01138	5.5E-06	0.79509	8.33E-09
cdh2$^{-/-}$; *fbn2b*$^{-/-}$	8.6E-06	2.1E-06	2.6E-07	1.9E-07	3.3E-06	0.79509	0.01138	NA	0.00077	0.5777	4.38E-07
cdh2$^{-/-}$; *fn1a*$^{-/-}$; *fn1b*$^{-/-}$	1.1E-08	3.9E-08	2.2E-09	1.6E-09	2.4E-07	0.00185	5.5E-06	0.00077	NA	0.00149	0.03143
cdh2-MO; *fbn2b*$^{-/-}$; *fn1a*$^{-/-}$; *fn1b*$^{-/-}$	0.00367	0.03208	0.00345	0.00245	0.0542	0.4134	0.79509	0.5777	0.00149	NA	8.29E-05
cdh2$^{-/-}$; *MZitgα5*$^{-/-}$	1.90E-10	9.18E-11	1.39E-11	1.09E-11	1.66E-09	1.02E-06	8.33E-09	4.38E-07	0.03143	8.29E-05	NA

Transverse Orientation

	wild type	*fbn2b*$^{-/-}$	*fn1a*$^{-/-}$; *fn1b*$^{-/-}$	*fbn2b*$^{-/-}$; *fn1a*$^{-/-}$; *fn1b*$^{-/-}$	*MZitgα5*$^{-/-}$	*cdh2*$^{-/-}$	*cdh2*-MO	*cdh2*$^{-/-}$; *fbn2b*$^{-/-}$	*cdh2*$^{-/-}$; *fn1a*$^{-/-}$; *fn1b*$^{-/-}$	*cdh2*-MO; *fbn2b*$^{-/-}$; *fn1a*$^{-/-}$; *fn1b*$^{-/-}$	*cdh2*$^{-/-}$; *MZitgα5*$^{-/-}$
wild type	NA	0.01396	0.43824	0.13213	0.06655	0.00065	5.5E-05	8.3E-05	2.5E-06	0.00091	4.27E-10
fbn2b$^{-/-}$	0.01396	NA	0.56834	0.98009	0.96535	0.05236	0.01428	0.01396	6.7E-05	0.27091	2.09E-08
fn1a$^{-/-}$; *fn1b*$^{-/-}$	0.43824	0.56834	NA	0.98009	0.98009	0.0065	0.00108	0.00118	1.3E-05	0.01673	5.22E-09
fbn2b$^{-/-}$; *fn1a*$^{-/-}$; *fn1b*$^{-/-}$	0.13213	0.98009	0.98009	NA	0.98009	0.08314	0.04596	0.03654	0.00011	0.38565	9.75E-07
MZitgα5$^{-/-}$	0.06655	0.96535	0.98009	0.98009	NA	0.01104	0.00162	0.00209	3E-05	0.02567	6.04E-09
cdh2$^{-/-}$	0.00065	0.05236	0.0065	0.08314	0.01104	NA	0.98009	0.98009	0.04652	0.98009	7.14E-04
cdh2-MO	5.5E-05	0.01428	0.00108	0.04596	0.00162	0.98009	NA	0.98009	0.01544	0.98009	4.04E-05
cdh2$^{-/-}$; *fbn2b*$^{-/-}$	8.3E-05	0.01396	0.00118	0.03654	0.00209	0.98009	0.98009	NA	0.02316	0.73419	9.73E-05
cdh2$^{-/-}$; *fn1a*$^{-/-}$; *fn1b*$^{-/-}$	2.5E-06	6.7E-05	1.3E-05	0.00011	3E-05	0.04652	0.01544	0.02316	NA	0.0021	0.77641
cdh2-MO; *fbn2b*$^{-/-}$; *fn1a*$^{-/-}$; *fn1b*$^{-/-}$	0.00091	0.27091	0.01673	0.38565	0.02567	0.98009	0.98009	0.73419	0.0021	NA	6.42E-06
cdh2$^{-/-}$; *MZitgα5*$^{-/-}$	4.27E-10	2.09E-08	5.22E-09	9.75E-07	6.04E-09	7.14E-04	4.04E-05	9.73E-05	0.77641	6.42E-06	NA

C PSM Cell Motion

	wild type	*fbn2b*$^{-/-}$	*fn1a*$^{-/-}$; *fn1b*$^{-/-}$	*fbn2b*$^{-/-}$; *fn1a*$^{-/-}$; *fn1b*$^{-/-}$	*MZitgα5*$^{-/-}$	*cdh2*$^{-/-}$	*cdh2*-MO	*cdh2*$^{-/-}$; *fbn2b*$^{-/-}$	*cdh2*$^{-/-}$; *fn1a*$^{-/-}$; *fn1b*$^{-/-}$	*cdh2*-MO; *fbn2b*$^{-/-}$; *fn1a*$^{-/-}$; *fn1b*$^{-/-}$	*cdh2*$^{-/-}$; *MZitgα5*$^{-/-}$
wild type	NA	0.9821	0.9821	0.93243	0.9821	0.46035	0.9821	0.9821	0.7539	0.9821	7.54E-03
fbn2b$^{-/-}$	0.9821	NA	0.95907	0.26556	0.83918	0.03333	0.9821	0.9821	0.08001	0.78343	3.08E-04
fn1a$^{-/-}$; *fn1b*$^{-/-}$	0.9821	0.95907	NA	0.9821	0.9821	0.7583	0.9821	0.9821	0.87027	0.9821	1.99E-02
fbn2b$^{-/-}$; *fn1a*$^{-/-}$; *fn1b*$^{-/-}$	0.93243	0.26556	0.9821	NA	0.9821	0.9821	0.8454	0.83918	0.9821	0.9821	4.60E-01
MZitgα5$^{-/-}$	0.9821	0.83918	0.9821	0.9821	NA	0.89513	0.9821	0.9821	0.9821	0.9821	6.34E-02
cdh2$^{-/-}$	0.46035	0.03333	0.7583	0.9821	0.89513	NA	0.24665	0.23383	0.9821	0.95907	9.59E-01
cdh2-MO	0.9821	0.9821	0.9821	0.8454	0.9821	0.24665	NA	0.9821	0.49903	0.9821	3.21E-03
cdh2$^{-/-}$; *fbn2b*$^{-/-}$	0.9821	0.9821	0.9821	0.83918	0.9821	0.23383	0.9821	NA	0.47415	0.9821	3.02E-03
cdh2$^{-/-}$; *fn1a*$^{-/-}$; *fn1b*$^{-/-}$	0.7539	0.08001	0.87027	0.9821	0.9821	0.9821	0.49903	0.47415	NA	0.9821	0.84101
cdh2-MO; *fbn2b*$^{-/-}$; *fn1a*$^{-/-}$; *fn1b*$^{-/-}$	0.9821	0.78343	0.9821	0.9821	0.9821	0.95907	0.9821	0.9821	0.9821	NA	9.65E-02
cdh2$^{-/-}$; *MZitgα5*$^{-/-}$	7.54E-03	3.08E-04	1.99E-02	4.60E-01	6.34E-02	9.59E-01	3.21E-03	3.02E-03	0.84101	9.65E-02	NA

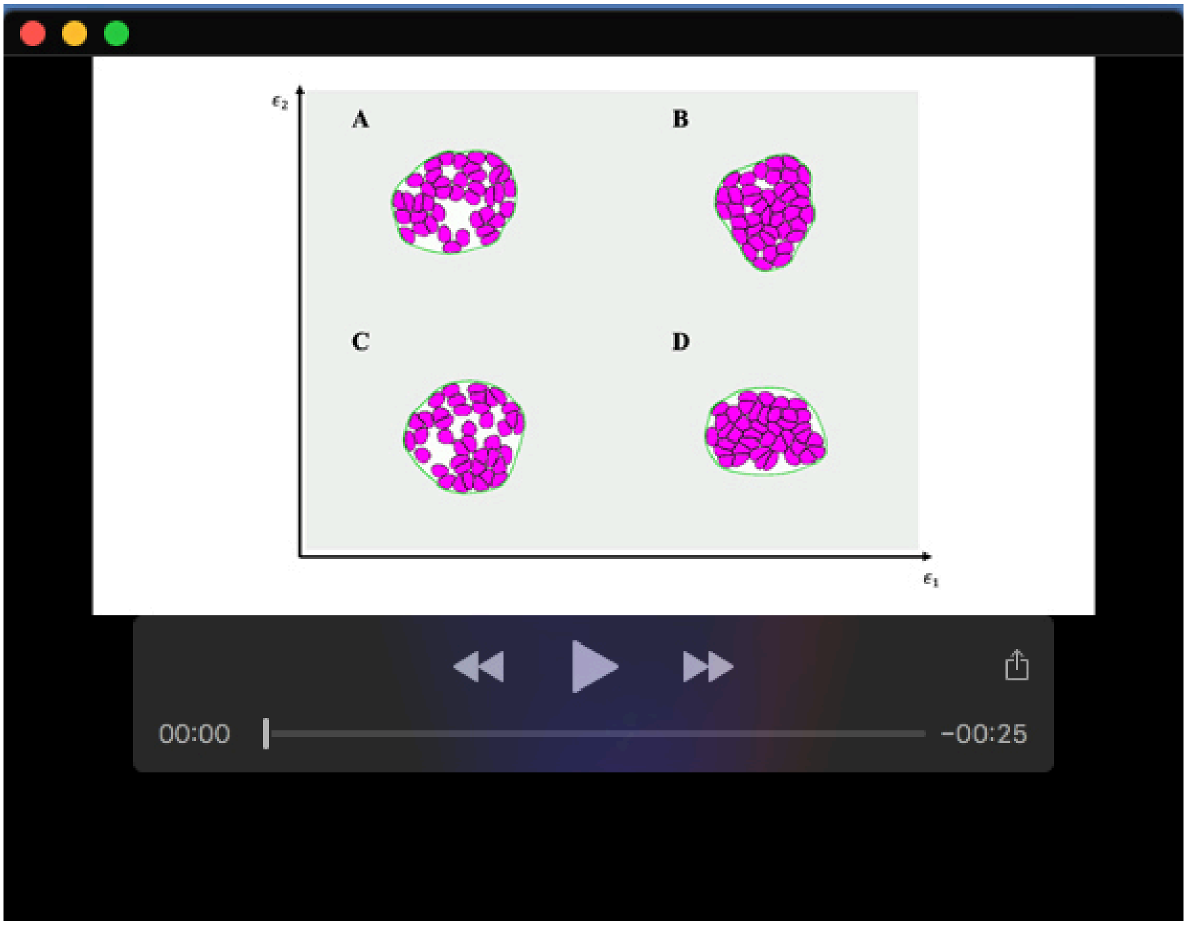

Movie 1. Simulation Movies without Cadherin 2 Repression of Integrin α5. Movies from the DEM simulations with parameters selected to represent the **(A)** *integrin α5* mutant, **(B)** wild type, **(C)** *integrin α5*; *cadherin 2* double mutant, and **(D)** *cadherin 2* mutant. Cells are shown in magenta, with the tissue boundary in green.

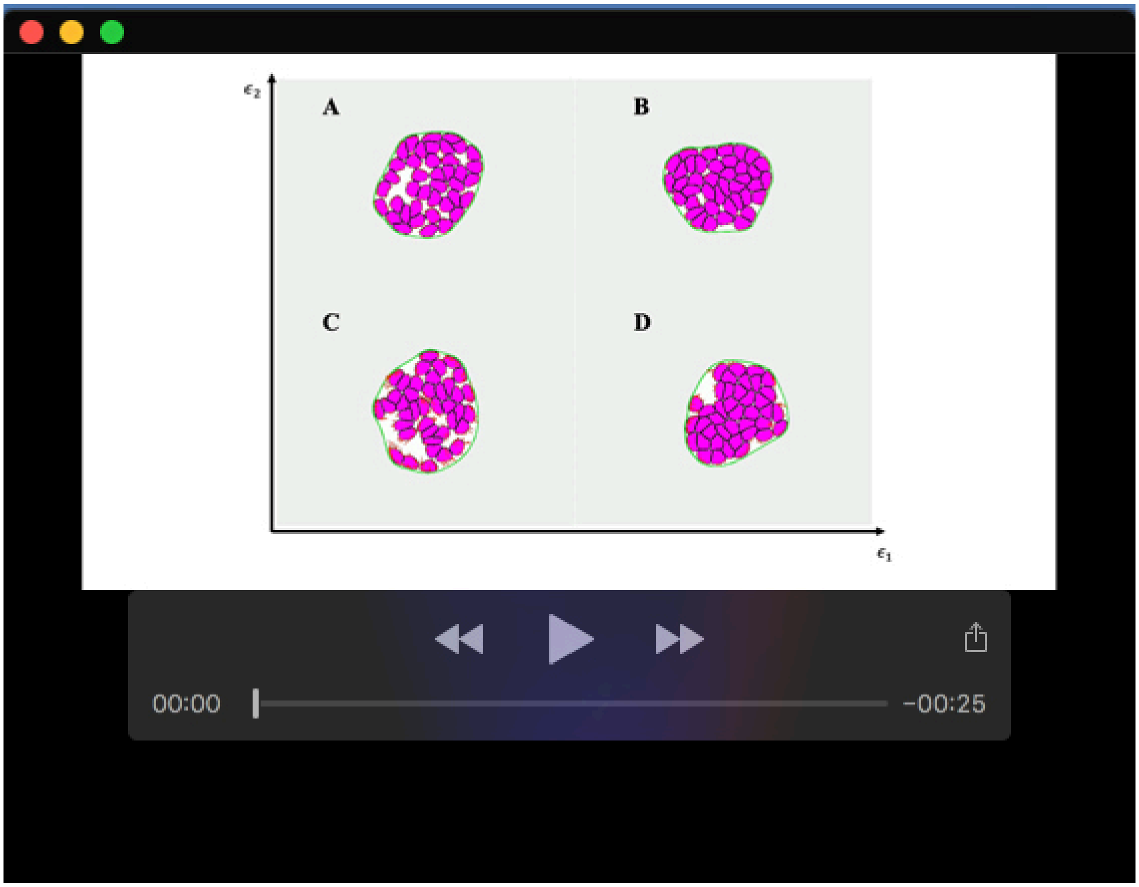

Movie 2. Simulation Movies with Cadherin 2 Repression of Integrin α5. Movies from the DEM simulations with parameters selected to represent the **(A)** *integrin α5* mutant, **(B)** wild type, **(C)** *integrin α5; cadherin 2* double mutant, and **(D)** *cadherin 2* mutant, incorporating an additional energy term for enhanced activation of Integrin α5 in the absence of Cadherin 2. Cells are shown in magenta, with the tissue boundary in green. Red lines represent cell-ECM bonds via additional activation of Integrin α5.
